# The mitochondrial unfolded protein response in human microglia disrupts neuronal–glial communication and promotes senescence

**DOI:** 10.1038/s41593-026-02320-1

**Published:** 2026-06-26

**Authors:** Maria Jose Perez J, Alicia Lam, Christin Weissleder, Federico Bertoli, Hariam Raji, Mariella Bosch, Ivan Nemazanyy, Stefanie Kalb, Mohammed Kehili, Insa Hirschberg, Dario Brunetti, Indra Heckenbach, Morten Scheibye-Knudsen, Michela Deleidi

**Affiliations:** 1 Mechanisms and Therapy of Genetic Brain Diseases, Institut Imagine, Paris, France; 2 Aligning Science Across Parkinson’s (ASAP) Collaborative Research Network, Chevy Chase, MD USA; 3 Hertie Institute for Clinical Brain Research, University of Tübingen, Tübingen, Germany; 4 Platform for Metabolic Analyses, Structure Fédérative de Recherche Necker, INSERM US24/CNRS UAR 3633, Paris, France; 5 Department of Clinical Sciences and Community Health, Excellence Department 2023–2027, University of Milan, Milan, Italy; 6 Unit of Medical Genetics and Neurogenetics, Fondazione IRCCS Istituto Neurologico ‘Carlo Besta’, Milan, Italy; 7 Department of Cellular and Molecular Medicine, Center for Healthy Aging, University of Copenhagen, Copenhagen, Denmark; 8 Present Address: Max Planck Institute of Immunobiology and Epigenetics, Freiburg, Germany

**Keywords:** Neuroimmunology, Molecular neuroscience, Mitochondria, Microglia

## Abstract

Mitochondria have evolved a specialized mitochondrial unfolded protein response (UPR^mt^) to maintain proteostasis and promote recovery under stress. Studies in simple organisms have shown that UPR^mt^ activation in glial cells supports proteostasis through beneficial non-cell-autonomous communication with neurons. However, the role of mitochondrial stress responses in the human brain remains unclear. To address this gap, we investigated the cell-type-specific effects of mitochondrial proteotoxic stress using human induced pluripotent stem cell-derived neuronal and glial cultures, as well as brain organoids. Here we show that mitochondrial proteotoxic stress induces metabolic rewiring in human microglia, marked by depletion of S-adenosylmethionine and lipid remodeling, ultimately leading to a senescent phenotype. Using human neuronal–glial tricultures and microglia-containing brain organoids, we identified the specific contributions of microglia to brain senescence and mitochondrial stress-driven neurodegenerative processes. UPR^mt^ activation disrupts microglial communication with neighboring cells, triggering inflammatory signaling and impairing proteostasis. Together, these findings reveal how impaired mitochondrial proteostasis alters intercellular networks and identify a critical role for the UPR^mt^ in neurodegenerative disease pathogenesis.

## Abstract (web summary)

Mitochondrial proteotoxic stress rewires methionine and lipid metabolism in human microglia, driving cellular senescence and disrupting neuronal–glial communication, revealing a mechanism linking mitochondrial dysfunction to brain aging and neurodegeneration.

## Main

Defects in proteostasis and protein aggregation are hallmarks of aging and neurodegenerative diseases. Mitochondria have evolved a specialized unfolded protein response (UPR^mt^) to maintain proteostasis and facilitate recovery under stress by inducing genes encoding mitochondrial chaperones and proteases^1,2^. Studies in *Caenorhabditis elegans* have demonstrated that UPR^mt^ activation can extend lifespan through metabolic adaptations and mitochondrial recovery programs^3,4^. Although these findings highlight protective roles for the UPR^mt^ in simple organisms, their relevance to the mammalian brain remains unclear. Neurons are long-lived, postmitotic cells that rely on tightly regulated mitochondrial quality control to maintain cellular homeostasis, raising key questions about how mitochondrial proteostasis is controlled in the aging brain and whether UPR^mt^ activation is beneficial or detrimental within neuronal and glial networks.

Age-dependent changes in UPR^mt^ regulation suggest a declining capacity to sense mitochondrial stress. For instance, activity of the mitochondrial protease LONP1 decreases with age, and PITRM1 expression is reduced in the aging brain and in Alzheimer’s disease (AD) patients^5,6^. Although UPR^mt^ activation can alleviate neurodegenerative phenotypes^7–9^, evidence also indicates that it may contribute to age-related brain disorders^10,11^. Although initially protective against mitochondrial protein misfolding, chronic UPR^mt^ activation may impair neuronal function and provoke maladaptive immune responses^12,13^. Specifically, the UPR^mt^ induces genes linked to mitochondrial repair and innate immunity^12,13^, promoting chronic inflammation—a hallmark of neurodegeneration.

In simple organisms, UPR^mt^ signaling can occur in a non-cell-autonomous manner, with neurons inducing UPR^mt^ activation in distant tissues. Recent studies in *C.* *elegans* further show that astrocyte-like glial cells can sense mitochondrial stress and initiate protective signaling that supports neuronal proteostasis^14^. These findings suggest that mitochondrial stress responses propagate across tissues through integrated intercellular signaling networks^15–17^. However, how mitochondrial proteotoxic stress is regulated in a cell-type-specific manner in the mammalian brain, and how it shapes intercellular communication, remain largely unexplored.

Here we define the cell-type-specific roles of the UPR^mt^ in the human brain. Using human induced pluripotent stem cell (iPSC)-derived monocultures, tricultures and disease-associated microglia-brain (MgBr) assembloids, we investigate how distinct cell types initiate and transmit mitochondrial proteotoxic stress signals and how these processes impact intercellular communication.

## Results

### Pharmacological inhibition of LONP1 differentially induces UPR^mt^ activation and protein aggregation in human iPSC-derived neurons and glial cells

**Fig. 1:**
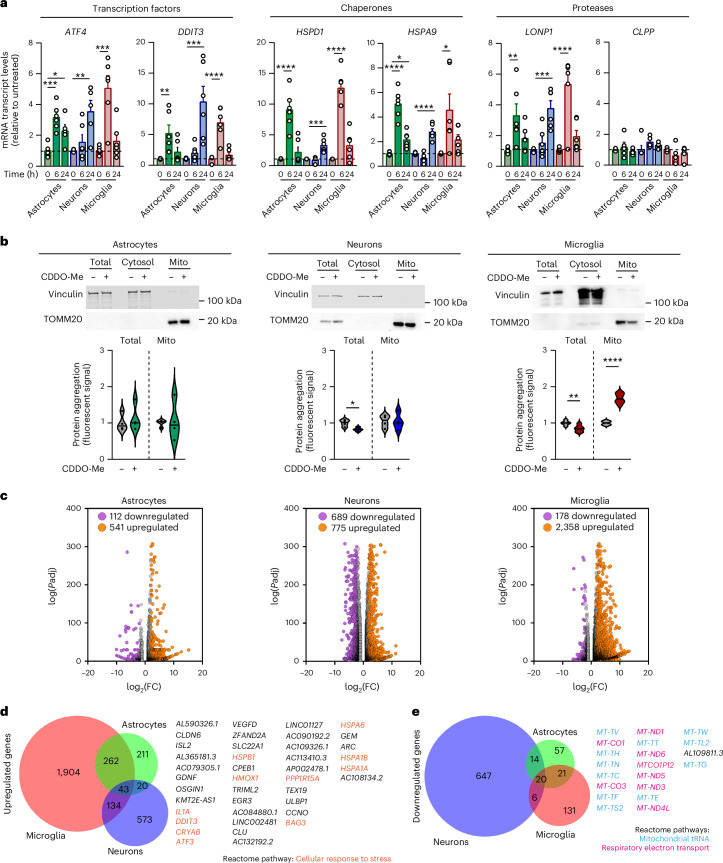
Pharmacological inhibition of LONP1 in human iPSC-derived neurons and glia induces cell-type-specific protein misfolding and activation of the UPR^mt^. All experiments were performed using control iPSC line C1. **a**, mRNA expression of UPR^mt^ genes in human iPSC-derived astrocytes, neurons and microglia treated with 1 µM CDDO-Me for 6 h or 24 h. Data are normalized to untreated controls (gray) within each cell type. Mean ± s.e.m.; one-way ANOVA with Bonferroni post hoc correction. *ATF4*: *P = 0.0229, **P = 0.004, ***P = 0.0003 (astrocytes) and 0.0002 (microglia), *DDIT3*: **P = 0.0097, ***P = 0.0009 and ****P < 0.0001; *HSPD1*: ***P = 0.0003 and ****P < 0.0001; *HSPA9*: *P = 0.0434 (astrocytes), 0.0117 (microglia), and ****P < 0.0001; *LONP1*: **P = 0.0083, ***P = 0.0002 and ****P < 0.0001; *n* = 6 independent experiments. Horizontal dashed line indicates the control-normalized expression level. **b**, Upper panel: representative immunoblots of total, cytosolic (vinculin) and mitochondrial (TOMM20) fractions from neurons, astrocytes and microglia before and after 1 µM CDDO-Me treatment (6 h). Lower panel: PROTEOSTAT fluorescence intensity in total and mitochondrial fractions. Violin plots show individual values and medians, normalized to untreated controls within each condition. Unpaired two-tailed *t*-test; *P = 0.0162, **P = 0.0060, ****P < 0.0001; *n* = 4 (astrocytes, neurons), *n* = 6 (microglia) independent experiments. **c**, Volcano plots of DEGs in astrocytes, neurons and microglia under control conditions and after CDDO-Me treatment. Differential expression was determined using DESeq2 with Benjamini–Hochberg correction. Genes with FC ≥ 2 and FDR ≤ 0.05 are highlighted. **d**,**e**, Venn diagrams showing shared upregulated (**d**) and downregulated (**e**) DEGs across cell types. Transcript isoforms were consolidated to single gene entries for counting. Top enriched Reactome pathways among shared DEGs are indicated. Cytosol, cytosolic; Mito, mitochondrial. Source data

We differentiated wild-type human iPSCs into cortical neurons, astrocytes and microglia (Supplementary Fig. 1a–h) and treated them with the LONP1 inhibitor C-28 methyl ester of 2-cyano-3,12-dioxo-olean-1,9(11)-dien-28-oic acid (CDDO-Me)^18^ to induce mitochondrial protein misfolding and activate the UPR^mt^ (Fig. 1a and Supplementary Fig. 2a–c). Microglia and astrocytes exhibited early UPR^mt^ activation at 6 h post-treatment, whereas peak activation in neurons occurred at 24 h (Fig. 1a). LONP1 inhibition caused mitochondrial fragmentation and decreased membrane potential across all cell types (Supplementary Fig. 2d). Next, we examined the cell-type-specific effects on proteostasis using the PROTEOSTAT assay. Whereas astrocytes and neurons showed no detectable protein accumulation, microglia exhibited a selective increase in mitochondria-associated misfolded proteins after CDDO-Me treatment (Fig. 1b). Costaining of intact cells with PROTEOSTAT and the mitochondrial markers TOMM20 and HSP60 confirmed the preferential association of misfolded proteins with mitochondria in microglia (Supplementary Fig. 2e).

### LONP1 inhibition elicits divergent stress responses in human iPSC-derived neurons and glia

Bulk RNA sequencing (RNA-seq) at peak UPR^mt^ activation (Fig. 1c and Supplementary Data 1) revealed robust induction of UPR^mt^-associated genes^19^ and a broad HSF1-mediated heat shock response across cell types (Fig. 1d and Supplementary Fig. 3a,b), consistent with quantitative PCR with reverse transcription (qRT–PCR) data (Fig. 1a) and previous reports in mouse embryonic fibroblasts^20^. In contrast, downregulated genes were predominantly mitochondrial-encoded (Fig. 1e), reflecting reduced mtDNA content and disrupted mitochondrial RNA metabolism in all cell types (Supplementary Fig. 3c–g).

Neurons and astrocytes mounted a compensatory proteostatic response marked by increased chaperone expression, activation of the endoplasmic reticulum UPR, enhanced autophagy and translational repression (Supplementary Fig. 3h). In contrast, microglia activated the UPR^mt^ while upregulating components of the translational machinery (Supplementary Fig. 3h), a signature previously linked to age-associated integrated stress response (ISR) adaptations^21–24^. LONP1 inhibition also induced cell-type-specific apoptotic gene programs, with increased *BAX* and *CASP8* and reduced *BCL2* expression in neurons, but elevated *BCL2* in glia (Supplementary Fig. 3h,i). Despite these transcriptional changes, no increase in cell death was detected in any cell type (Supplementary Fig. 3j).

Pathway-level analyses further revealed distinct stress responses across cell types (Supplementary Fig. 4a,b and Supplementary Data 2). Astrocytes preferentially engaged stress-adaptive and innate immune signaling pathways (Supplementary Fig. 4a,b). Functionally, this molecular shift did not compromise cellular stability; astrocytes maintained redox homeostasis, as indicated by stable 2,7-dichlorofluorescein (DCF) levels (Supplementary Fig. 4c), and showed no significant change in GFAP protein expression (Supplementary Fig. 4d). Neurons exhibited a pronounced oxidative stress response, characterized by the activation of *NFE2L2*-mediated transcriptomic programs (Supplementary Fig. 4a,b) and a functional increase in reactive oxygen species (ROS) (Supplementary Fig. 4e). Although markers of tau and α-synuclein pathology remained unchanged (Supplementary Fig. 4f), this stress state resulted in physiological impairments, evidenced by a significant reduction in neuronal excitability and calcium signaling (Supplementary Fig. 4g,h). In contrast, microglia displayed a distinct transcriptional profile characterized by senescence-associated remodeling, including epigenetic alterations, changes in nuclear architecture and activation of senescence-related inflammatory pathways (Supplementary Fig. 4a,b). Together, these data indicate that whereas astrocytes adapt and neurons undergo functional impairment without overt degeneration, microglia transition toward a dysfunctional, senescence-like state in response to mitochondrial proteotoxic stress.

### LONP1 inhibition induces a senescent phenotype in human iPSC-derived microglia

**Fig. 2:**
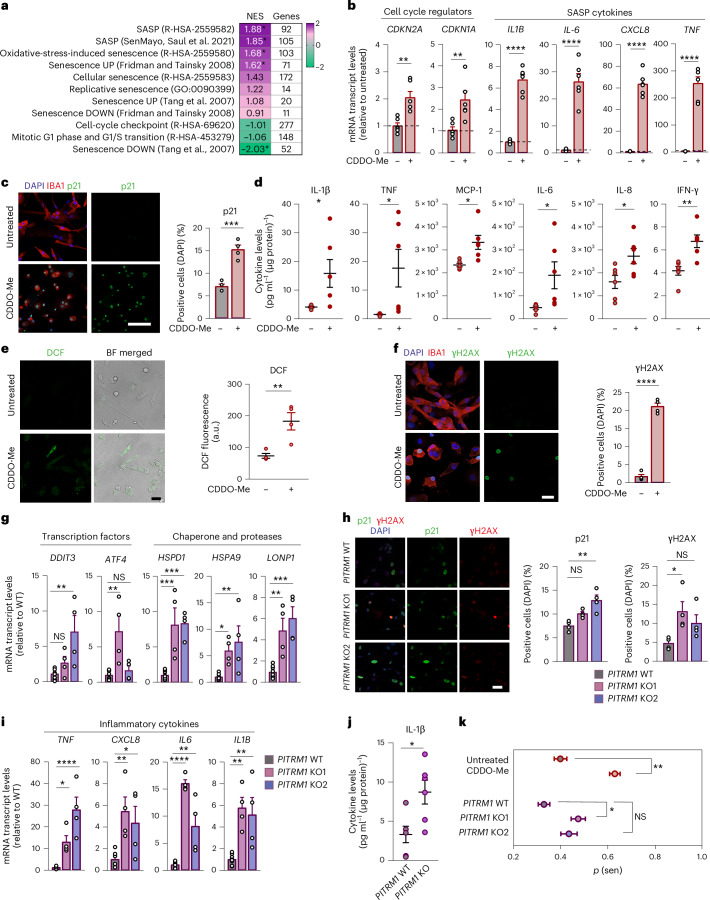
Mitochondrial proteotoxic stress induces a senescent phenotype in human iPSC-derived microglia. **a**–**k**, Microglial senescence was examined in pharmacological (CDDO-Me treatment in line C1; **a**–**f**) and genetic (*PITRM1-*KO; **g**–**k**) models associated with UPR^mt^ activation. **a**, Heatmap showing enrichment of senescence-associated gene sets in RNA-seq data from microglia treated with CDDO-Me^26,77,78^. NES were calculated by GSEA relative to untreated controls. Upregulation is shown in purple and downregulation in green. Asterisk, FDR ≤ 0.05. **b**, mRNA expression of *CDKN2A*, *CDKN1A* and SASP cytokines (*IL1B*, *IL-6*, *CXCL8*, *TNF*) following CDDO-Me treatment. Data are normalized to untreated controls (gray). Mean ± s.e.m.; unpaired two-tailed *t*-test. *CDKN2A* **P = 0.0036; *CDKN1A* **P = 0.0016; *IL1B*, *IL-6*, *CXCL8*, *TNF*, ****P < 0.0001; *n* = 6 independent experiments. **c**, Left: representative confocal images of p21 (green) in human iPSC-derived microglia (red: IBA1; blue: DAPI). Scale bar, 50 µm. Right: quantification of the percentages of p21^+^/DAPI^+^. Mean ± s.e.m.; unpaired two-tailed *t*-test; ***P = 0.0004; *n* = 4 independent experiments. **d**, Cytokine levels in supernatants from untreated or CDDO-Me-treated (6 h) microglia. Mean ± s.e.m.; unpaired two-tailed *t*-test. *P = 0.0356 (IL-1β), 0.0336 (TNF), 0.0105 (MCP-1), 0.0381 (IL-6), 0.0283 (IL-8) and **P = 0.0033; *n* = 6 independent experiments. **e**, Left: representative confocal images of DCF-based ROS measurements in control and CDDO-Me-treated microglia. Scale bar, 10 µm. Right: quantification of DCF fluorescence intensity. Mean ± s.e.m.; unpaired two-tailed *t*-test; ** *P* = 0.0085; *n* = 4 independent experiments. **f**, Left: representative γH2AX immunostaining in control and CDDO-Me-treated microglia (green: γH2AX; red: IBA1; blue: DAPI). Scale bar, 10 µm. Right: quantification of γH2AX^+^/DAPI^+^ cells. Mean ± s.e.m.; unpaired two-tailed *t*-test; **** *P* < 0.0001; *n* = 4 independent experiments. **g**, mRNA expression of UPR^mt^ genes in *PITRM1*-WT and two independent *PITRM1*-KO clones (KO1, KO2). Data are normalized to WT controls (gray). Mean ± s.e.m.; one-way ANOVA with Bonferroni post hoc correction. *DDIT3*, **P = 0.0032; *ATF4*, **P = 0.0013; *HSPD1*, ***P = 0.0010 (KO1), 0.0008 (KO2); *HSPA9*, *P = 0.0484, **P = 0.0092; *LONP1*, **P = 0.0030, ***P = 0.0003; NS, not significant; *n* = 4 independent biological replicates per KO clone and its matched WT. **h**, Left: representative p21 (green) and γH2AX (red) staining in *PITRM1*-WT and *PITRM1*-KO microglia (DAPI, blue). Scale bar, 10 µm. Right: quantification of the percentage of p21^+^/DAPI^+^ and γH2AX^+^/DAPI^+^. Mean ± s.e.m.; one-way ANOVA with Bonferroni post hoc correction. *P = 0.0338, **P = 0.0014; *n* = 4 independent experiments. **i**, SASP gene expression (*TNF*, *CXCL8*, *IL-6*, *IL1B*) in *PITRM1*-WT and KO microglia (KO1 and KO2). Mean ± s.e.m.; normalized to WT controls (gray); one-way ANOVA with Bonferroni post hoc correction. *TNF*, *P = 0.0190, ****P < 0.0001; *CXCL8*, *P = 0.0373; **P = 0.0072; *IL6*, **P = 0.0010, ****P < 0.0001; *IL1B*, **P = 0.0020 (KO1), 0.0059 (KO2); *n* = 4 independent biological replicates per KO clone and its matched WT. **j**, IL-1β levels in supernatants of *PITRM1*-WT and *PITRM1*-KO2 microglia. Mean ± s.e.m.; unpaired two-tailed *t*-test; *P = 0.0152; *n* = 6 independent experiments. **k**, Predicted senescence scores in untreated, CDDO-Me-treated, *PITRM1*-WT and *PITRM1*-KO human iPSC-derived microglia. Mean ± s.e.m.; unpaired two-tailed *t*-test (CDDO-Me) and one-way ANOVA with Bonferroni post hoc correction (*PITRM1*); *P = 0.0258, **P = 0.0078; NS = not significant; *n* = 4 independent experiments. Source data

To examine the relationship between mitochondrial proteotoxic stress and microglial senescence, we performed gene-set enrichment analysis (GSEA) using 11 published senescence-associated gene signatures^25–28^. CDDO-Me-treated iPSC-derived microglia and neurons showed significant positive enrichment of gene sets upregulated in senescence and negative enrichment of those typically downregulated (Fig. 2a and Supplementary Fig. 5a). qRT–PCR and immunostaining revealed that only CDDO-Me-treated microglia upregulated *CDKN2A* (p16) and *CDKN1A* (p21), together with increased expression and secretion of senescence-associated secretory phenotype (SASP) cytokines, including IL-1β, IL-6, IL-8 and TNF (Fig. 2b–d and Supplementary Fig. 5b–d). These findings were validated independently in two additional iPSC lines (Supplementary Fig. 5e–i).

Flow cytometric analysis following Hoechst 33342 staining showed that astrocytes underwent a modest redistribution from G1 to S phase, consistent with a stress-induced reactive state (Supplementary Fig. 6a–c). In contrast, iPSC-derived microglia and neurons exhibited a largely nonproliferative profile with minimal G2 representation that was unchanged by treatment with CDDO-Me or the senescence inducer etoposide (Supplementary Fig. 6d,e), indicating that iPSC-derived microglia are largely terminally differentiated^29^ and mitochondrial stress induces senescence without cell cycle re-entry. Additional hallmarks confirmed the senescent phenotype. Senescence-associated β-galactosidase (SA-β-gal) activity, already detectable under basal conditions^30^, was increased following CDDO-Me treatment (Supplementary Fig. 6f). This was accompanied by elevated ROS production, γH2AX-positive nuclei (Fig. 2e,f), increased NAD^+^ consumption and enhanced PARP1 activity (Supplementary Fig. 6g,h), consistent with DNA damage-associated senescence^31–34^.

To exclude off-target effects, *LONP1* was silenced using shRNA (Supplementary Fig. 7a). Knockdown recapitulated p21 induction, γH2AX accumulation and increased IL-8 secretion (Supplementary Fig. 7b,c). Similarly, pharmacological UPR^mt^ activation with gamitrinib-TPP (GTPP)^35^ or *ATF5* overexpression induced senescence markers and SASP gene expression (Supplementary Fig. 7d–g). Together, these data demonstrate that mitochondrial proteotoxic stress and UPR^mt^ activation are sufficient to drive a selective senescence-like program in human microglia.

### Mitochondrial proteotoxic stress induces a transcriptional state associated with early pathological microglial activation

Although senescence markers normalized following CDDO-Me withdrawal (Supplementary Fig. 8a,b), senescence-associated stress can imprint long-lasting molecular changes^29^. To test whether mitochondrial stress alters immune reactivity, we challenged CDDO-Me-pretreated iPSC-derived microglia with LPS. Pretreated cells released more IL-1β but less TNF and IL-6 (Supplementary Fig. 8c), consistent with age-associated immune remodeling and persistent inflammatory reprogramming^36,37^.

To assess disease relevance, we compared the transcriptional profile of CDDO-Me-treated microglia with human microglial clusters from the Human Microglia Atlas (HuMicA)^38^. Enrichment analysis revealed significant overlap with early pathological states linked to inflammation, amyloid pathology and cellular stress in aging and neurodegeneration (Supplementary Fig. 8d,e and Supplementary Data 1). The strongest similarities were observed for programs marked by immediate early genes, proinflammatory signaling and heat shock responses, reported in AD and amyloid-exposed microglia^39^. The CDDO-Me signature also overlapped with phagocytic and phospho-tau-associated microglial states, while diverging from homeostatic and proliferative programs^39–41^ (Supplementary Fig. 8d). Visualization of the top intersecting genes showed reduced microglial identity alongside activation of inflammatory, UPR and heat shock pathways (Supplementary Fig. 8e). Partial overlap with disease-inflammatory macrophages^42^ further supports a maladaptive stress trajectory. Together, these data indicate that acute mitochondrial proteotoxic stress primes microglia toward an early pathological activation state characteristic of human aging and neurodegeneration.

### Disease-associated defects in mitochondrial proteostasis induce a senescence-like phenotype in human iPSC-derived microglia

To validate the disease relevance of our findings, we examined models deficient in *PITRM1*—a mitochondrial metallopeptidase that degrades mitochondrial targeting sequences after their cleavage from proteins imported into mitochondria^43^. Its impairment activates the UPR^mt^ in yeast, human iPSC-derived neurons and brain organoids^8,44^, whereas loss-of-function mutations cause a slowly progressive autosomal recessive neurodegenerative disorder with cognitive decline^45,46^. PITRM1 also contributes to amyloid-β (Aβ) clearance, and its dysfunction results in Aβ accumulation^8,45,47,48^. Accordingly, *Pitrm1*^+/−^ mice develop progressive neurological symptoms and brain Aβ_1‒42_ accumulation resembling AD pathology^45^. Notably, *Pitrm1*^*+*^*/*^*−*^ mice displayed an age-dependent increase in SA-β-gal activity in the cortex and hippocampus. At 15 months, this was accompanied by an elevated number of p21-positive microglia and TNF levels (Supplementary Fig. 9a–d), consistent with microglial senescence.

To assess cell-autonomous effects in human cells, we generated *PITRM1* wild-type (WT) and knockout (KO) iPSC-derived microglia. *PITRM1* deficiency did not impair differentiation, as shown by comparable numbers of IBA1- and PU.1-positive cells (Supplementary Fig. 9e), but robustly activated the UPR^mt^ (Fig. 2g). *PITRM1*-KO microglia upregulated *CDKN2A* (p16) and *CDKN1A* (p21) at transcript and protein levels (Fig. 2h and Supplementary Fig. 9f), displayed increased SASP cytokine expression (*IL1B*, *IL6*, *CXCL8*, *TNF*) and IL-1β secretion, and showed elevated oxidative stress, γH2AX-positive nuclei and NAD^+^ consumption (Fig. 2i,j and Supplementary Fig. 9g–i). In contrast, *PITRM1*-deficient astrocytes and neurons did not exhibit increased p21 expression or DNA damage (Supplementary Fig. 9j). Together, these findings demonstrate that genetic defects in mitochondrial proteostasis associated with human neurodegenerative disease selectively drive a senescence-like program in microglia.

### Morphology-based deep learning identifies UPR^mt^-driven senescence in human iPSC-derived microglia

To address the challenge of detecting senescence in nondividing cells^49,50^, we leveraged characteristic nuclear features of senescent cells—including increased nuclear area, reduced convexity and altered aspect ratio—and applied a machine learning-based classifier to identify senescent microglia. This approach has been validated to distinguish senescence from quiescence^51^. Both CDDO-Me-treated and *PITRM1*-KO microglia exhibited significantly higher predicted senescence scores compared with controls (Fig. 2k). Notably, CDDO-Me-treated microglia showed higher senescence scores, larger nuclear area and reduced convexity and aspect ratio relative to *PITRM1*-KO cells, consistent with a more uniform and pronounced senescence phenotype induced by acute pharmacological stress.

### UPR^mt^ activation dysregulates lipid metabolism in human iPSC-derived microglia

**Fig. 3:**
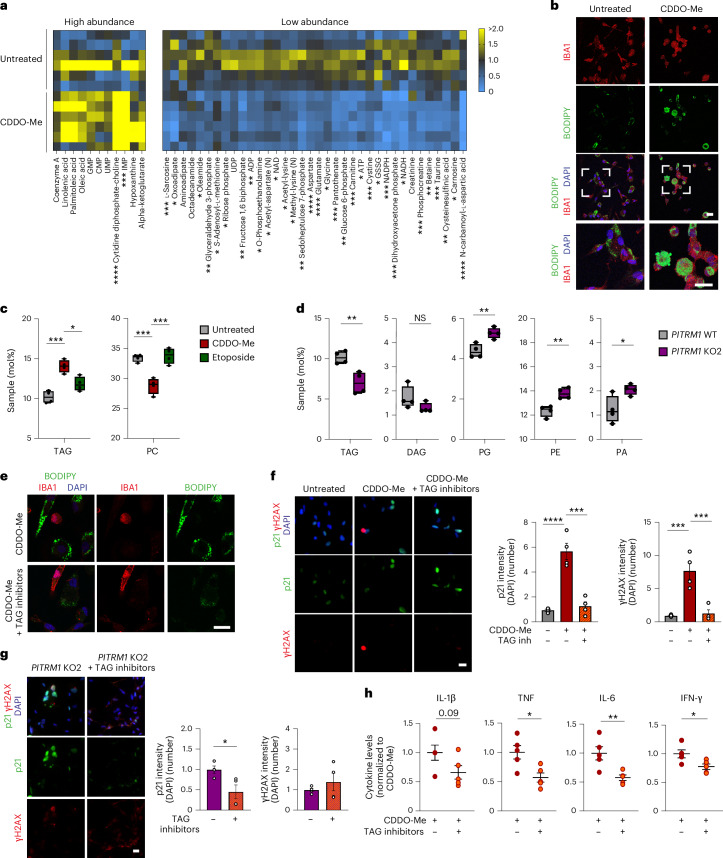
UPR^mt^ disrupts lipid metabolism in human iPSC-derived microglia. All experiments were performed using iPSC line C2 unless otherwise indicated. **a**, Heatmap of the top most significantly altered metabolites in control and CDDO-Me-treated microglia. Metabolites are ranked according to relative abundance in CDDO-Me-treated cells compared to controls (left: higher abundance; right: lower abundance). Yellow indicates increased and blue decreased metabolite levels. Data are normalized to control; unpaired two-tailed *t*-test; *P = 0.0366 (oxoadipate), 0.0351 (oleamide), 0.0212 (*S*-adenosyl-ʟ-methionine), 0.0290 (ribose phosphate), 0.0269 (*O*-phosphoethanolamine), 0.0186 (acetyl-aspartate), 0.02 (NAD), 0.0161 (acetyl-lysine), 0.0206 (methyl-lysine), 0.0102 (glycine), 0.0176 (ATP), 0.0270 (GSSG), 0.0107 (NADH) and 0.0175 (carnosine); **P = 0.0046 (glyceraldehyde-3-phosphate), 0.0030 (fructose-1,6-biphosphate), 0.0098 (ADP), 0.0016 (sedoheptulose-7-phosphate), 0.0010 (glucose-6-phosphate), 0.0017 (betaine) and 0.0030 (cysteine sulfinic acid); ***P = 0.0003 (IMP), 0.0007 (ʟ-sarcosine), 0.0009 (pantothenate), 0.005 (carnitine), 0.0002 (cystine), 0.0006 (NADPH), 0.0003 (dihydroxyacetone phosphate), 0.0002 (phosphocreatine) and 0.0009 (taurine); ****P < 0.0001; *n* = 6 independent experiments. **b**, Representative confocal images of IBA1 (red), BODIPY (green) and DAPI (blue) staining in microglia. Scale bars, 10 µm. Images are representative of at least two independent experiments. **c**, LC–MS quantification of TAG and PC (mol%) in untreated, CDDO-Me-treated and etoposide-treated microglia. Center line, median; box limits, 25th–75th percentiles; whiskers, minimum–maximum. One-way ANOVA with Bonferroni post hoc correction. TAG: *P = 0.0118, ***P = 0.0003; PC, ***P = 0.0008 (untreated versus CDDO-Me) and 0.0005 (CDDO-Me versus etoposide); *n* = 4 independent experiments. **d**, LC–MS analysis of TAG, DAG, phosphatidylglycerol (PG), PE, and phosphatidic acid (PA) (mol%) in *PITRM1*-WT and *PITRM1*-KO2 microglia. Center line, median; box limits, 25th–75th percentiles; whiskers, minimum–maximum. Unpaired two-tailed *t*-test. TAG, **P = 0.0063; PG, **P = 0.0060; PE, **P = 0.0037; PA *P = 0.0320; *n* = 4 independent experiments. **e**, Representative confocal images of IBA1 (red), BODIPY (green) and DAPI (blue) staining in microglia treated with CDDO-Me with or without TAG inhibitors (5 µM). Scale bar, 10 µm. Representative of at least two independent experiments. **f**, Left: representative images of p21 (green) and γH2AX (red) staining in untreated, CDDO-Me-treated and CDDO-Me + TAG inhibitors (5 µM) conditions; blue: DAPI. Scale bar, 10 µm. Right: quantification of p21 and γH2AX fluorescence intensity in DAPI^+^ cells, normalized to control. Mean ± s.e.m.; one-way ANOVA with Bonferroni post hoc correction. p21, ***P = 0.0001, ****P < 0.0001; γH2AX, ***P = 0.0006 (untreated versus CDDO-Me), 0.0009 (CDDO-Me versus CDDO-Me + TAG inhibitors); *n* = 4 independent experiments. **g**, Left: representative images of p21 (green) and γH2AX (red) staining in *PITRM1*-KO2 microglia with or without TAG inhibitors (10 µM); blue: DAPI. Scale bar, 10 µm. Right: quantification normalized to *PITRM1*-KO2. Mean ± s.e.m.; unpaired two-tailed *t*-test; *P = 0.0290; *n* = 4 independent experiments. **h**, Cytokine levels in supernatants from CDDO-Me-treated, and CDDO-Me + TAG inhibitors-treated microglia, normalized to CDDO-Me-treated. Mean ± s.e.m.; unpaired two-tailed *t-*test. TNF, *P = 0.0152; IL-6, **P = 0.0092; IFN-γ, *P = 0.0267; *n* = 5 independent experiments. Source data

To explore the link between mitochondrial stress responses, lipid metabolism and senescence^14,52,53^, we performed targeted mass spectrometry-based metabolic profiling in control and CDDO-Me-treated iPSC-derived microglia (Supplementary Data 3). Principal component analysis (PCA) and hierarchical clustering revealed marked metabolic shifts upon UPR^mt^ activation (Fig. 3a and Supplementary Fig. 10a).

Integration of transcriptomic and metabolomic data identified enrichment of lipid pathways, including glycerophospholipid and glycerolipid metabolism, with decreased dihydroxyacetone phosphate and O-phosphoethanolamine and increased CDP-choline^54^ (Fig. 3a and Supplementary Fig. 10b). Twenty lipid metabolism genes were dysregulated, affecting phosphatidylcholine (PC), phosphatidate, phosphatidylethanolamine (PE), phosphatidylserine and CDP-diacylglycerol (DAG) pathways (Supplementary Fig. 10b).

BODIPY staining showed altered lipid droplet (LD) distribution in CDDO-Me-treated microglia (Fig. 3b). Lipidomics revealed decreased PC alongside triacylglycerol (TAG) accumulation, consistent with a shift toward lipid storage (Fig. 3c and Supplementary Fig. 10c), whereas *PITRM1*-KO microglia exhibited reduced TAG and DAG, and increased membrane phospholipids (Fig. 3d and Supplementary Fig. 10d). Inhibition of TAG synthesis by DGAT1/2 reduced LD formation and senescence markers (p21 and γH2AX) in both models (Fig. 3e–g and Supplementary Fig. 10e), and decreased TNF, IL-6 and IFN-γ secretion by CDDO-Me-treated microglia (Fig. 3h), indicating that TAG synthesis is required for UPR^mt^-induced senescence.

### UPR^mt^ activation dysregulates methionine metabolism in human iPSC-derived microglia

**Fig. 4:**
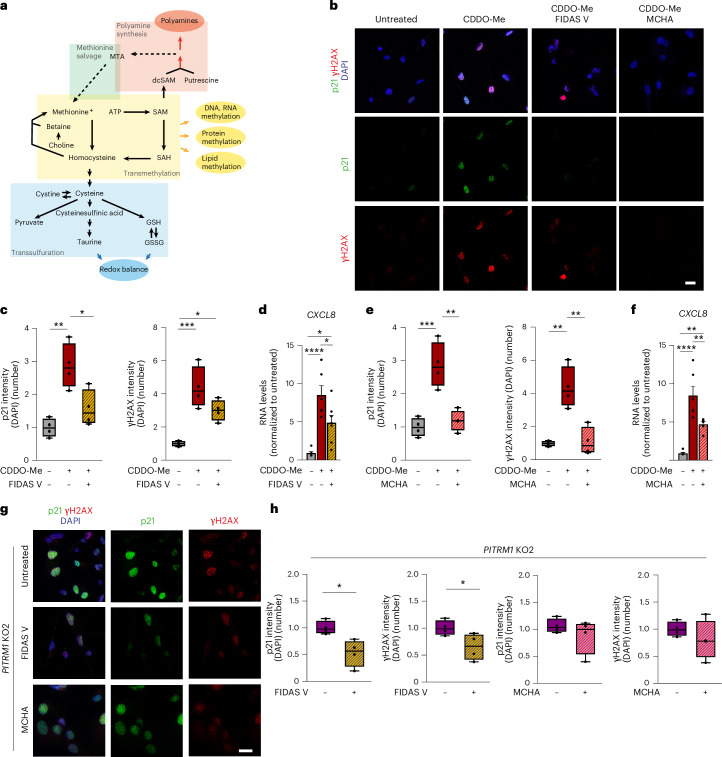
Reducing SAM availability attenuates the senescent phenotype in human iPSC-derived microglia. All experiments were performed using iPSC line C2, unless otherwise indicated. **a**, Schematic of key metabolites in methionine and cysteine metabolism, including transmethylation, methionine salvage, polyamine synthesis and transsulfuration pathways. **b**, Representative confocal images of p21 (green) and γH2AX (red) in microglia untreated or treated with 1 µM CDDO-Me alone or combined with 25 µM FIDAS V or 250 µM MCHA for 6 h. Nuclei were stained with DAPI (blue). Scale bar, 10 µm. **c**, Quantification of p21^+^/DAPI^+^ and γH2AX^+^/DAPI^+^ fluorescence in untreated, CDDO-Me-treated and CDDO-Me + FIDAS V groups. Data normalized to untreated controls. Center line, median; box, 25th–75th percentiles; whiskers, minimum–maximum. One-way ANOVA with Bonferroni post hoc correction. p21, *P = 0.0223, **P = 0.0023; γH2AX, *P = 0.0209; ***P = 0.0007; *n* = 4 independent experiments. **d**, *CXCL8* mRNA levels under cotreatment conditions. Data normalized to untreated controls, mean ± s.e.m.; one-way ANOVA with Bonferroni post hoc correction; *P = 0.0133 (CDDO-Me versus CDDO-Me + FIDAS V), 0.0305 (untreated versus CDDO-Me + FIDAS V); ****P < 0.0001; *n* = 7 independent experiments. **e**, Quantification of p21^+^/DAPI^+^ and γH2AX^+^/DAPI^+^ fluorescence in untreated, CDDO-Me-treated and CDDO-Me + MCHA groups. Data normalized to untreated controls. Center line, median; box, 25th–75th percentiles; whiskers, minimum–maximum. One-way ANOVA with Bonferroni post hoc correction. p21, **P = 0.0018, ***P = 0.0008; γH2AX **P = 0.0012 (untreated versus CDDO-Me), 0.0016 (CDDO-Me versus CDDO-Me + MCHA); *n* = 4 independent experiments. **f**, *CXCL8* mRNA levels in cotreated cells. Data normalized to untreated controls, mean ± s.e.m.; one-way ANOVA with Bonferroni post hoc correction; **P = 0.0069 (untreated versus CDDO-Me + MCHA), 0.0078 (CDDO-Me versus CDDO-Me + MCHA) and ****P < 0.0001; *n* = 6 independent experiments. **g**, Representative confocal images of p21 (green) and γH2AX (red) in *PITRM1*-KO2 microglia, untreated or treated with 25 µM FIDAS V or 250 µM MCHA for 6 h. Nuclei: DAPI (blue). Scale bar, 10 µm. **h**, Quantification of p21^+^/DAPI^+^ and γH2AX^+^/DAPI^+^ fluorescence in *PITRM1*-KO (KO2) microglia under indicated treatments. Data normalized to untreated controls. Center line, median; box, 25th–75th percentiles; whiskers, minimum–maximum. Unpaired two-tailed *t*-test, *P = 0.0147 (p21), 0.0460 (γH2AX); *n* = 4 independent experiments. Source data

We performed metabolite set enrichment analysis to identify metabolic pathways altered in CDDO-Me-treated human iPSC-derived microglia. Amino acid metabolism pathways were significantly enriched, including the malate-aspartate shuttle, lysine degradation and β-alanine metabolism (Supplementary Fig. 11a). Folate and cysteine metabolism were dysregulated markedly, with decreased levels of glycine, sarcosine, cysteine sulfinic acid, betaine, taurine, cystine and S-adenosylmethionine (SAM) (Figs. 3a and 4a).

Consistent with impaired glutathione synthesis, total glutathione (GSH) levels were decreased, although the GSH/glutathione disulfide (GSSG) ratio was increased (Supplementary Fig. 11b), suggesting compensatory redox adaptation. Transcriptomic analysis revealed upregulation of methionine adenosyltransferase II (*MAT2A*), which catalyzes SAM synthesis from methionine and ATP, and cystathionine β-synthase (*CBS*), a key regulator of homocysteine metabolism (Supplementary Data 1). These data support coordinated rewiring of methionine and redox metabolism during UPR^mt^ activation and link these changes to microglial senescence.

To test the contribution of oxidative stress, we treated cells with the mitochondria-targeted antioxidant mitoquinone mesylate (MitoQ). MitoQ reduced p21 and DNA damage and restored *HSPD1*, *DDIT3* and *CXCL8* expression to baseline in CDDO-Me-treated microglia (Supplementary Fig. 11c,d), consistent with ROS acting upstream of UPR^mt^^55^. However, MitoQ failed to rescue p21 or γH2AX levels in *PITRM1*-KO microglia (Supplementary Fig. 11e), indicating that antioxidant treatment alone is insufficient to reverse the senescence phenotype in the context of persistent mitochondrial proteostasis defects.

Metabolic profiling of *PITRM1*-KO microglia revealed similar disruption of methionine, betaine, and taurine pathways (Supplementary Fig. 11f,g and Supplementary Data 3). Both CDDO-Me-treated and *PITRM1*-KO microglia exhibited significant reductions in L-sarcosine, cystine and SAM levels (Fig. 4a and Supplementary Fig. 11f). Together, these data identify impaired methionine metabolism as a shared metabolic signature of UPR^mt^-driven microglial senescence across pharmacological and genetic models.

### Targeting SAM metabolism attenuates the senescence phenotype in human iPSC-derived microglia

To determine whether methionine metabolism contributes to UPR^mt^-driven senescence, we modulated SAM availability in CDDO-Me-treated human iPSC-derived microglia. Supplementation with SAM-HCl increased DNA damage and p21 immunofluorescence compared with CDDO-Me alone (Supplementary Fig. 12a–c). SAM-HCl also elevated *HSPD1* transcripts without affecting *DDIT3* and enhanced *CXCL8* expression (Supplementary Fig. 12d), indicating partial UPR^mt^ activation together with amplification of the SASP.

Given the upregulation of *MAT2A* and reduced SAM levels upon CDDO-Me treatment, we hypothesized that increased SAM flux into downstream pathways, including polyamine synthesis, contributes to senescence. Inhibition of SAM synthesis with the MAT2A inhibitor FIDAS V significantly reduced p21 protein levels and *CXCL8* transcripts in CDDO-Me-treated microglia (Fig. 4b–d and Supplementary Fig. 12e). Similarly, blockade of spermidine synthesis with MCHA decreased DNA damage, p21, and *CXCL8* expression (Fig. 4b,e,f). Neither FIDAS V nor MCHA altered UPR^mt^ markers (*HSPD1, DDIT3*) (Supplementary Fig. 12f), indicating that attenuation of senescence occurred downstream of UPR^mt^ activation. In *PITRM1*-KO microglia, FIDAS V reduced p21 and DNA damage, whereas MCHA showed a similar trend (Fig. 4g,h and Supplementary Fig. 12g,h). Although TAG inhibition did not restore SAM levels, both FIDAS V and MCHA reduced LD accumulation (Supplementary Fig. 12i,j). Together, these findings suggest that SAM availability regulates lipid remodeling and represents a key metabolic regulator of UPR^mt^-driven microglial senescence.

### UPR^mt^ activation induces a microglia-specific senescence phenotype in iPSC-derived tricultures

**Fig. 5:**
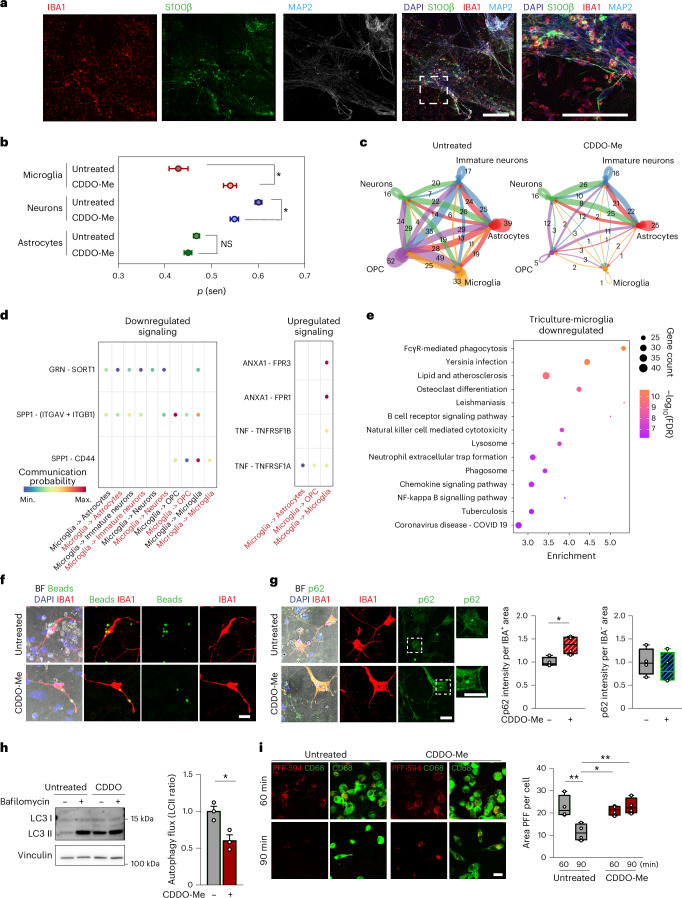
Mitochondrial proteotoxic stress impairs cell–cell communication and misfolded protein clearance in human iPSC-derived microglia in mono- and tricultures. All experiments were performed using iPSC line C2. **a**, Representative confocal images of control iPSC-derived tricultures showing microglia (IBA1, red), astrocytes (S100β, green) and neurons (MAP2, white). Nuclei were stained with DAPI (blue). Scale bars, 50 µm. **b**, Predicted senescence scores (*p* (sen)) of untreated and CDDO-Me-treated human iPSC-derived microglia, neurons and astrocytes in tricultures. Mean ± s.e.m.; unpaired two-tailed *t*-test compared with untreated controls in each cell type, *P = 0.0106 (microglia), 0.0138 (neurons); *n* = 3 independent experiments. **c**,**d**, Cell–cell communication in tricultures analyzed using CellChat. **c**, Circle plots showing number of putative ligand–receptor (L–R) interactions in control versus CDDO-Me-treated cells. Edge width represents communication strength. **d**, Bubble plot depicting up- and downregulated L–R pairs in microglia within the triculture. Colors indicate communication probability, comparing CDDO-Me treatment with untreated control. Black font: interactions under control; red font: interactions under CDDO-Me. Significance determined by permutation test (*n* = 100); bubbles represent significant L–R interactions (nominal *P* < 0.01). **e**, Bubble plot showing downregulated Reactome pathways in the microglial cluster. Bubble diameter indicates gene count; color represents −log_10_(FDR). **f**, Fluorescence imaging of Alexa Fluor 488-labeled beads (green) and IBA1 (red) in untreated and CDDO-Me-treated microglia. Nuclei stained with DAPI (blue). BF, brightfield. Scale bar, 10 µm. Images are representative of at least two independent experiments. **g**, Left: immunostaining for p62 (green) and IBA1 (red) in untreated and CDDO-Me-treated tricultures. Nuclei: DAPI (blue). Scale bars, 10 µm. Right: quantification of p62 intensity in IBA1^+^ and IBA1^−^ areas. Data normalized to untreated controls. Center line, median; box, 25th–75th percentiles; whiskers, minimum–maximum; unpaired two-tailed *t*-test, *P = 0.0167; *n* = 4 independent experiments. **h**, Western blot of LC3-II in microglia, untreated or treated with CDDO-Me, with or without bafilomycin (4 h). Autophagic flux quantified as the ratio of LC3-II in bafilomycin-treated versus untreated cells, normalized to untreated control. Mean ± s.e.m.; unpaired two-tailed *t*-test, *P = 0.0192; *n* = 3 independent experiments. **i**, Left: confocal images of microglia untreated or treated with CDDO-Me, stained for CD68 (green) and α-synuclein preformed fibrils (PFF-594, red) at 60- and 90-min post-treatment. Nuclei: DAPI (blue). Scale bar, 10 µm. Right: quantification of PFF-594 area relative to total cell area. Center line, median; box, 25th–75th percentiles; whiskers, minimum–maximum; two-way ANOVA with Bonferroni post hoc correction, *P = 0.0170, **P = 0.0058 (untreated 60 min versus untreated 90 min), 0.0048 (untreated 90 min versus CDDO-Me 90 min); *n* = 4 independent experiments. Source data

To examine whether microglial senescence persists in a multicellular context, we established a human iPSC-derived triculture system composed of 30% neurons, 60% astrocytes and 10% microglia (Fig. 5a). Cultures were maintained for 7 days before treatment with 1 µM CDDO-Me for 6 h. Cell-type-specific senescence was quantified using nuclear morphology-based deep learning analysis (Fig. 5b). Consistent with monoculture experiments, CDDO-Me treatment selectively increased the predicted senescence score in microglia, whereas neurons and astrocytes showed reduced scores compared to controls (Fig. 5b), indicating a microglia-specific senescence response within the triculture environment. To validate these findings in a disease-relevant context, we analyzed *PITRM1*-KO tricultures. As observed following pharmacological induction, *PITRM1*-deficient microglia displayed significantly higher predicted senescence scores compared to neurons or astrocytes (Supplementary Fig. 13a). Together, these data demonstrate that mitochondrial proteostasis defects and UPR^mt^ activation drive a robust and cell-type-specific senescence program in human iPSC-derived microglia, even in a complex multicellular milieu.

### UPR^mt^ activation induces cell-type-specific metabolic dysregulation in iPSC-derived tricultures

To investigate intercellular mechanisms of UPR^mt^ activation, we performed single-cell RNA-seq in CDDO-Me-treated tricultures. UMAP integration and clustering identified 11 clusters, including two microglial, one immature neuronal, three neuronal, four astrocytic and one oligodendrocyte progenitor cluster (OPC) (Supplementary Fig. 13b–e and Supplementary Data 4). CDDO-Me did not alter cluster proportions (Supplementary Fig. 13f).

UCell analysis revealed lower basal UPR^mt^ activity in neurons than glia, with CDDO-Me increasing scores across all cell types (Supplementary Fig. 13g,h). Comparing monoculture and triculture differentially expressed genes (DEGs) identified 985 triculture-specific dysregulated genes (63 neuronal, 308 astrocytic and 614 microglial; Supplementary Fig. 13i and Supplementary Data 5), highlighting context-dependent transcriptional responses. Reactome analysis showed disruption of inflammatory pathways, including lipid and/or atherosclerosis and antiviral responses (Supplementary Data 6). UPR^mt^ activation induced cell-type-specific metabolic rewiring: microglia upregulated nucleotide sugar metabolism (R-HSA-00520, −01250), whereas neurons upregulated amino acid and one-carbon metabolism, as well as cysteine and/or methionine pathways (R-HSA-01230, −00260, −00220, −00250, −01240, −01200, −00670, −00270). These results indicate that glia-neuron crosstalk shapes inflammatory and metabolic responses to mitochondrial proteotoxic stress.

### Mitochondrial proteotoxic stress decreases predicted microglia-mediated cell–cell communication and phagocytic signaling in iPSC-derived tricultures

To define how UPR^mt^-driven senescence reshapes intercellular communication, we analyzed ligand–receptor interactions in the single-cell dataset using CellChat^56^. CDDO-Me treatment globally reduced both the number and strength of predicted interactions in tricultures, indicating an overall attenuation of cell–cell communication (Fig. 5c). Cell-type-resolved analysis revealed increased signaling among astrocytes and neuronal populations, but a marked reduction in autocrine and paracrine communication from microglia and OPCs (Supplementary Fig. 14a).

In microglia, CDDO-Me enhanced ANXA1–FPR1/3 and TNF-TNFRSF1A/1B signaling, consistent with increased inflammatory crosstalk toward astrocytes, OPCs and neighboring microglia (Fig. 5d). Conversely, microglial SPP1-(ITGAV^+^ITGB1) signaling to neurons, astrocytes, OPCs and microglia was reduced, as was SPP1–CD44 signaling to microglia and OPCs and GRN–SORT1 signaling to astrocytes, OPCs and neurons (Fig. 5d). Given the role of SPP1 in macrophage phagocytosis and microglial synapse engulfment^57^, these changes suggest impaired clearance-related communication.

Consistently, Reactome analysis of microglial DEGs revealed enrichment of phagocytosis-related pathways (Fcγ receptor (FcγR)-mediated phagocytosis, lysosome, phagosome), infection-response pathways, and lipid and atherosclerosis-related pathways (Fig. 5e and Supplementary Fig. 14b). Functional assays in tricultures showed unchanged latex bead uptake but significant accumulation of p62 in CDDO-Me-treated microglia, indicating defective autophagic processing (Fig. 5f,g). A similar phenotype was observed in tricultures containing *PITRM1*-KO microglia with wild-type neurons and astrocytes (Supplementary Fig. 14c,d), demonstrating that microglia-intrinsic mitochondrial stress is sufficient to impair clearance mechanisms. To determine whether synaptic remodeling was affected, we assessed PSD95 accumulation. CDDO-Me treatment and *PITRM1* deficiency both increased PSD95 signal within microglia and across cultures, without changes in C1QA levels (Supplementary Fig. 14e), consistent with impaired synaptic pruning^58^. In monoculture, bead uptake was reduced after CDDO-Me treatment (Supplementary Fig. 14f). LAMP1 intensity was increased significantly in IBA1-positive microglia, suggesting lysosomal stress or expansion (Supplementary Fig. 14g). Moreover, bafilomycin further reduced autophagic flux in CDDO-Me-treated cells (Fig. 5h and Supplementary Fig. 14h). Together, these findings demonstrate that mitochondrial proteotoxic stress induces a microglial senescence-like state characterized by impaired intercellular communication, reduced phagocytic signaling and autophagolysosomal dysfunction—features highly relevant to neurodegenerative disease contexts.

### UPR^mt^ -induced misfolded protein accumulation in senescent microglia is reversed by inhibition of polyamine synthesis

To examine the relevance of UPR^mt^-driven senescence to protein aggregation, we cotreated control iPSC-derived microglia with CDDO-Me and Alexa Fluor 594-labeled α-synuclein preformed fibrils (PFFs). Immunostaining at 60 min and 90 min revealed increased intracellular α-synuclein accumulation in CDDO-Me-treated microglia compared to controls, indicating impaired fibril clearance (Fig. 5i). *PITRM1*-KO microglia similarly exhibited elevated intracellular α-synuclein relative to isogenic controls (Supplementary Fig. 14i).

To test whether polyamine synthesis contributes to this phenotype, we cotreated microglia with α-synuclein PFFs and CDDO-Me in the presence or absence of the polyamine synthesis inhibitor MCHA. Live-cell imaging confirmed progressive α-synuclein accumulation in CDDO-Me-treated cells (Supplementary Fig. 14j). Notably, MCHA significantly reduced intracellular α-synuclein levels compared to CDDO-Me alone, and this effect persisted over time (Supplementary Fig. 14j). These findings demonstrate that UPR^mt^-induced misfolded protein accumulation in senescent microglia is dependent on SAM-driven polyamine metabolism and can be reversed by its inhibition.

### Microglial UPR^mt^ drives senescence and protein aggregation in MgBr assembloids

**Fig. 6:**
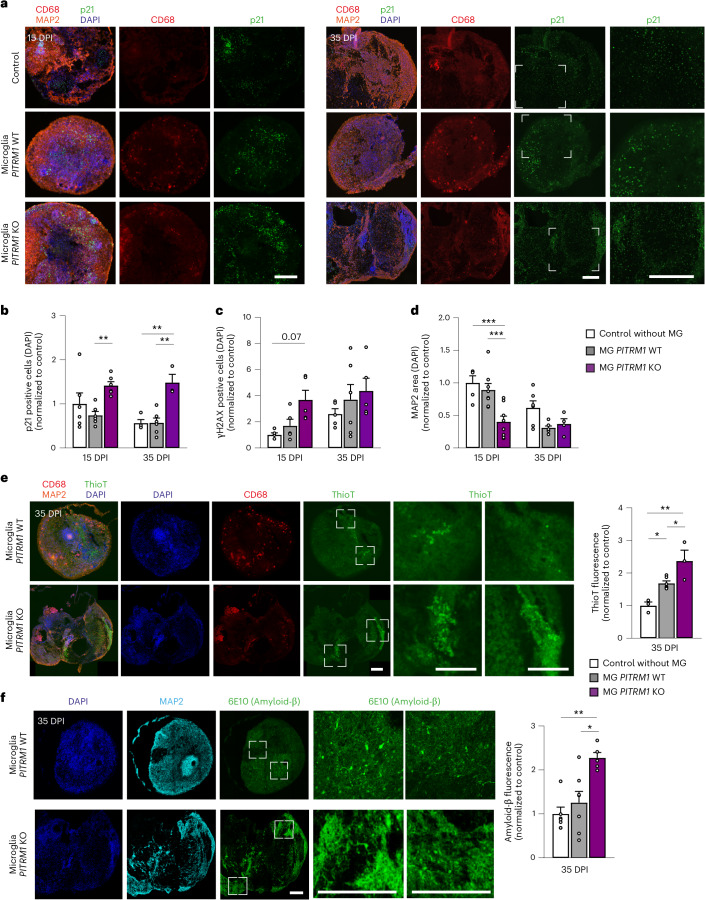
Mitochondrial proteotoxic stress in microglia induces senescence propagation and misfolded protein accumulation in MgBr assembloids. All experiments were performed using iPSC line C2 and *PITRM1-*KO2. **a**, Immunostaining for p21 (green), CD68 (red) and MAP2 (orange) in MgBr assembloids at 15 and 35 DPI. Nuclei were counterstained with DAPI (blue). High-magnification images of the sections are shown on the right. Scale bars, 25 µm. **b–d**, Quantification of p21^+^/DAPI^+^ cells (**b**), γH2AX^+^/DAPI^+^ cells (**c**) and MAP2/DAPI area (**d**) in whole sections at 15 and 35 DPI. Data are shown as mean ± s.e.m. and normalized to control organoids without microglia integration. Two-way ANOVA with Bonferroni post hoc correction between genotypes at each time point. Significant comparisons: (**b**) **P = 0.0069 (15 DPI, *PITRM1*-WT versus *PITRM1*-KO), 0.0054 (35 DPI, Control versus *PITRM1*-KO), 0.0029 (35 DPI, *PITRM1*-WT versus *PITRM1*-KO); (**d**) ***P = 0.0002 (15 DPI, Control versus *PITRM1*-KO) and 0.0004 (15 DPI, *PITRM1*-WT versus *PITRM1*-KO). Each *n* represents one individual organoid. For **b**, *n* = 6/4 Control, 6/6 WT and 6/3 KO at 15/35 DPI, respectively. For **c**, *n* = 5/5 Control, 5/6 WT and 5/5 KO. For **d**, *n* = 5/6 Control, 8/7 WT and 8/4 KO. **e**, Left: immunostaining for CD68 (red) and MAP2 (orange) in 35 DPI MgBr assembloids. Nuclei stained with DAPI (blue); protein aggregates detected with Thioflavin T (ThioT, green); high-magnification images are shown on the right. Scale bars, 25 µm. Right: quantification of ThioT fluorescence in whole sections. Mean ± s.e.m., normalized to control organoids without microglia. One-way ANOVA with Bonferroni post hoc correction; *P = 0.0402 (Control versus *PITRM1*-WT), 0.0409 (*PITRM1*-WT versus *PITRM1*-KO), **P = 0.0015; *n* = 3 Control, *n* = 6 WT, *n* = 3 KO individual organoids. **f**, Left: immunostaining for amyloid-β (6E10, green) and MAP2 (cyan) in 35 DPI MgBr assembloids. Nuclei stained with DAPI (blue). High-magnification images are shown on the right. Scale bars, 20 µm. Right: quantification of amyloid-β fluorescence in whole sections. Mean ± s.e.m.; normalized to control organoids without microglia integration. One-way ANOVA with Bonferroni post hoc correction; *P = 0.0136, **P = 0.0053. *n*: *n* = 5 Control, *n* = 7 WT, *n* = 5 KO individual organoids. Source data

To investigate the impact of microglial mitochondrial stress on brain senescence, we generated MgBr assembloids by integrating WT or *PITRM1*-KO microglia into WT cortical organoids at day 30 (Supplementary Fig. 15a,b). Microglia successfully integrated and persisted at 5, 15 and 35 days postintegration (DPI) (Supplementary Fig. 15c–e). Expression of endodermal, neuronal progenitor and mesodermal markers was comparable across conditions at 35 DPI (Supplementary Fig. 16a,b), indicating preserved lineage composition. In contrast, senescence markers revealed a progressive increase in p21 expression in organoids containing *PITRM1*-KO microglia, without changes in DNA damage markers (Fig. 6a–c and Supplementary Fig. 16c,d). Notably, MAP2 expression was significantly reduced at 35 DPI (Fig. 6d), suggesting impaired neuronal integrity associated with microglial mitochondrial stress. Assessment of amyloidogenic protein accumulation using thioflavin T demonstrated significantly increased fluorescence in *PITRM1*-KO MgBr assembloids at 35 DPI (Fig. 6e). Consistently, pathological Aβ species were elevated (Fig. 6f), whereas phosphorylated tau levels remained unchanged (Supplementary Fig. 16e,f), consistent with an early-stage pathology in which Aβ deposition precedes tau alterations^59–61^. Together, these findings indicate that microglial UPR^mt^ promotes a senescence-associated phenotype and accelerates amyloid accumulation in MgBr assembloids.

## Discussion

We define cell-type-specific roles for mitochondrial stress responses in the human brain and identify the UPR^mt^ as a key driver of microglial senescence. UPR^mt^ activation in human iPSC-derived microglia induces coordinated dysregulation of SAM metabolism and TAG-centered lipid remodeling, leading to impaired autophagolysosomal and phagocytic function, altered intercellular communication and compromised neuronal integrity. These findings link mitochondrial proteostasis defects to glia-driven brain aging and neurodegeneration.

Consistent with previous studies in mammalian and invertebrate systems, mitochondrial proteotoxic stress activated the UPR^mt^ in neurons, astrocytes and microglia, accompanied by reduced mtDNA content, decreased respiratory complex levels and increased chaperone expression^35,62^. However, glial cells, particularly microglia, exhibited earlier and more robust stress responses than neurons, suggesting an evolutionary specialization in which glia act as primary sensors, activating stress programs before neurons reach their own threshold^14,63^. Single-cell analyses in tricultures further revealed that astrocytes and microglia contribute disproportionately to mitochondrial stress signaling, suggesting glial specialization in stress sensing and response. Despite UPR^mt^ activation, microglia displayed a maladaptive program: downregulation of autophagy and proteasome genes combined with upregulation of translational machinery, reminiscent of ISR activation^24^. In contrast, neurons and astrocytes mounted compensatory proteostatic programs. Convergence of UPR^mt^ and ISR signaling in microglia may therefore promote a neurotoxic state.

Because defining senescence in postmitotic brain cells is challenging^28,30,64–66^, we combined transcriptional profiling, molecular analyses and machine learning-based nuclear morphology assessment to define a UPR^mt^-driven microglial senescence phenotype. This state is characterized by nuclear alterations, ISR-enriched transcriptional signatures, LD accumulation and defective phagocytosis, features absent in neurons or astrocytes under identical stress conditions, underscoring the selective vulnerability of microglia.

Previous research in simple organisms indicates that mitochondrial stress enhances neuronal and astrocyte signaling and thereby contributes to brain homeostasis^14,17^. However, our findings suggest a more divergent response in humans. Although glial cells in *C.* *elegans* and humans share fundamental roles, human glial cells exhibit greater complexity and specialization in neural function, development and regeneration^67^. Functionally, UPR^mt^ activation enhanced neuron-astrocyte communication but reduced microglial signaling, including decreased SPP1- and GRN-mediated phagocytic pathways. This was accompanied by increased TNF-dependent inflammatory signaling and impaired clearance of misfolded proteins. In MgBr assembloids, integration of *PITRM1*-KO microglia led to progressive p21 induction, reduced MAP2 expression and increased amyloid accumulation, supporting a causal contribution of microglial mitochondrial stress to brain senescence and early neurodegenerative pathology, potentially by driving the SASP^68^.

Lipid remodeling emerged as a central mechanism. Mitochondrial stress induced profound alterations in TAG metabolism, glycerophospholipid remodeling, oxidative stress and depletion of methionine-derived metabolites including SAM. We show that UPR^mt^ activation triggers a transition toward a dysfunctional state reminiscent of LD-accumulating microglia and senescent microglia, observed near tau and amyloid pathology in the aging brain^69,70^. This lipid accumulation is not merely a byproduct but a driver of dysfunction, as DGAT1/2 inhibition rescued both lipid storage and senescence markers. Despite divergent TAG levels in pharmacological versus genetic models, both converged on a senescent phenotype, suggesting that lipid flux rather than absolute lipid content is a critical determinant of cellular health^71–74^. This aligns with lipid dysregulation as a conserved hallmark of senescence across species^53,75^.

We further identify SAM metabolism as a key regulator of this process. Limiting SAM consumption or synthesis attenuated lipid accumulation and senescence markers, consistent with previous evidence that elevated SAM can promote UPR^mt^ activation^76^. This metabolic dependency helps explain distinct stages of the stress response: although targeting mitochondrial ROS with MitoQ prevented senescence during pharmacological stress, it failed to rescue the defects driven by chronic genetic UPR^mt^ activation. Together, these findings support a two-phase model of microglial decline: an initial ROS-dependent activation phase followed by a chronic, self-sustaining phase driven by the coordinated dysregulation of SAM and lipid metabolism. Ultimately, these data position one-carbon metabolism as the central node coupling mitochondrial stress to lipid remodeling and the progression toward senescence.

This evidence supports a convergent mechanism in which mitochondrial stress and ISR signaling cooperate to drive a lipid-mediated neurotoxic microglial state. Our findings extend the concept of ISR-marked neurodegenerative microglial subsets^24^, which in AD models promote ‘dark microglia’ and exacerbate synapse loss and proteinopathy. We show that targeting specific branches of lipid metabolism may attenuate microglia-driven senescence and neurodegeneration. Notably, the mitochondrial proteostasis stress signature identified in iPSC-derived microglia overlaps with disease-associated clusters in the aging and AD brain, including ISR activation, early stress responses and impaired phagocytic signaling, indicating that these maladaptive programs are active in human neurodegeneration.

Together, our findings highlight the context-dependent and cell-type-specific nature of UPR^mt^ activation. Although mitochondrial stress responses can support neuronal proteostasis^8,9,14^, sustained microglial UPR^mt^ activation drives a lipid-mediated, ISR-associated senescent state that disrupts neuronal homeostasis. These results argue against indiscriminate modulation of mitochondrial stress pathways and instead support microglia-targeted strategies, potentially through lipid nanoparticle delivery or selective modulation of downstream metabolic branches, to mitigate aging- and neurodegeneration-associated pathology.

## Methods

### Human iPSCs

Three healthy control lines were used in the study: C1 and C2 (refs. ^8,79,80^), and the commercial line C3 (Kolf2.1J, RRID: CVCL_B5P3; Jackson Laboratory). C1 and C2 iPSC lines were derived with informed consent and approved by the Ethics Committee of the Medical Faculty and University Hospital Tübingen (Ethikkommission der Medizinischen Fakultät am Universitätsklinikum Tübingen, 199/2011BO1). *PITRM1*-KO lines were generated from C2 (ref. ^8^). All lines were routinely tested for mycoplasma (Venor GeM Classic, Minerva Biolabs Inc., cat. no. 11-1250) and maintained in mTeSR Plus (STEMCELL Technologies; cat. no. 100-0276).

### Human iPSC differentiation

Human iPSCs were differentiated into cortical neurons, astrocytes and microglia using established stepwise protocols (Supplementary Methods). Cortical neurons were generated via neural progenitors^8,81^ and used after 21 days in vitro. Astrocytes were derived from neural progenitors^82^ and used between passages three and ten. Microglia were produced through embryoid-body-based hematopoietic specification^80,83^ followed by 10 days of terminal differentiation. Cells were maintained as monocultures or combined to generate multicellular systems. Tricultures were established by adding astrocytes and microglia sequentially to neurons at a defined ratio (neurons:astrocytes:microglia = 3:1:1) and maintained in maturation medium supplemented with microglial survival factors before experiments.

### Microglia-containing brain assembloids

Cortical organoids were generated^84^ and later integrated with predifferentiated iPSC-derived microglia to produce microglia-containing brain (MgBr) assembloids. Assembloids were maintained under orbital shaking and analyzed at defined days postintegration. Detailed differentiation conditions, media compositions and assembly procedures are described in Supplementary Methods.

### Drug treatments

Human iPSC-derived neurons, astrocytes and microglia were exposed to increasing concentrations of CDDO-Me (CAS number 218600-53-4, Cayman Chemical, cat. no. 11883) for the durations indicated in the figure legends. iPSC-derived microglia were exposed to GTPP (CAS number 1131626-47-5; MedChemExpress, cat. no. 50-196-7948). Additional treatments included ultrapure lipopolysaccharides from *Escherichia* *coli* O111:B4 (LPS-EB Ultrapure; InvivoGen, cat. no. tlrl-eblps), SAM-HCl (CAS No. 86867-01-8; Sigma–Aldrich, cat. no. CIAH9ABED0C7), FIDAS V (CAS No. 1391934-98-7; MedChemExpress, cat. no. HY-136144), MCHA (Sigma–Aldrich, cat. no. CDS019932), MitoQ (CAS No. 845959-50-4; MedChemExpress, cat. no. HY-100116AR) and etoposide (CAS No. 33419-42-0; MedChemExpress, cat. no. HY-13629). For TAG inhibition, cells were treated with the DGAT1 inhibitor PF-04620110 (CAS No. 1109276-89-2; MedChemExpress, cat. no. HY-13009) and the DGAT2 inhibitor PF-06424439 (CAS No. 1469284-78-3; MedChemExpress, cat. no. HY-108341).

### *LONP1* knockdown in iPSC-derived microglia

iPSC-derived microglia were transduced with lentiviral particles encoding an shRNA targeting human *LONP1* (sh*LONP1*; TRCN0000046793, MISSION shRNA library, Sigma–Aldrich) or a nontargeting control shRNA (VectorBuilder, cat. no. VB010000-0005mme). The sh*LONP1* construct (target sequence: CCAGTGTTTGAAGAAGACCAA) was cloned into the pLKO.1-puro backbone. Lentiviral particles were generated in HEK293T cells (RRID: CVCL_0063) by cotransfection of the shRNA plasmid with psPAX2 (RRID: Addgene_12260) and pMD2.G (RRID: Addgene_12259) using TransIT-X2 (Mirus Bio, cat. no. MIR 6003) according to the manufacturer’s protocol. Viral titers were normalized based on p24 capsid levels quantified with Lenti-X GoStix Plus (Takara Bio, cat. no. 631280), and equal amounts of virus were applied directly to microglial cultures without additional transfection reagents. Cells were collected for analysis at 24 h, 48 h and 72 h post-transduction.

### ATF5-inducible human iPSCs

iPSCs were transduced with lentiviral vectors encoding doxycycline-inducible constructs for *ATF5* overexpression (VectorBuilder, ID: VB241111-1111fuf) or an empty vector control (VectorBuilder, ID: VB201122-1083bzq), both containing a neomycin resistance cassette. Plasmid sequences and maps are available at Zenodo (10.5281/zenodo.20391259). Clonal selection was performed with G418 (150 µg ml^−1^; Thermo Scientific, cat. no. 10131035) for 2 weeks. Stable clones were expanded and used for experiments without further antibiotic selection.

### Mouse studies

All experiments were conducted in accordance with European Directive 2010/63/EU and Italian law (D. Lgs. n. 26/2014) and approved by the Italian Ministry of Health (authorization 483/2023-PR). C57BL/6N-Atm1Brd *Pitrm1*^+^/tm1a(KOMP)Wtsi mice (allele ID: MGI:5085349), kindly provided by the Wellcome Trust Sanger Institute, were derived from the KOMP-CSD ES cell line (RRID: MMRRC_060296-UCD). Animals were housed in groups of three per cage (22 ± 2 °C, 12 h light–dark cycle, 60% relative humidity) with access to food (rodent diet 4RF25, Mucedola, Italy) and water ad libitum. Two groups of female mice (6 months and 15 months; 6 *Pitrm1*^+/+^ and 6 *Pitrm1*^+/−^) were used. Brains were either formalin-fixed for histology or half-brains were fresh-frozen for Western blot or embedded in OCT (Tissue-Tek, Sakura Finetek, cat. no. 4565). Cryosections (20 µm) were stored in antifreeze solution.

### Immunofluorescence and live-cell imaging

Cultures and tissue sections were fixed with paraformaldehyde (Sigma–Aldrich, cat. no. 1004968350), permeabilized and blocked before incubation with primary and fluorescent secondary antibodies. Nuclei were counterstained with 4′,6-diamidino-2-phenylindole (DAPI; Sigma–Aldrich, cat. no. D9542-10MG). Lipid droplets and protein aggregates were visualized using BODIPY (Thermo Scientific, cat. no. D3922) and thioflavin T (Sigma‒Aldrich, cat. no. T3516), respectively, where indicated. Images were acquired by Leica TCS SP8 confocal microscope (×60/1.4 numerical aperture (NA) oil objective) and quantitative analyses, including nuclear marker quantification, fluorescence intensity measurements and colocalization, were performed using CellProfiler (Version 4.2.6, RRID: SCR_007358) and Fiji (Version 2.7.0, RRID:SCR_002285).

Cells were incubated at 37 °C with fluorescent probes to assess mitochondrial membrane potential (TMRM and MitoTracker Green; Invitrogen, cat. nos. T668 and M46750), intracellular calcium levels (Fluo-4 AM; Invitrogen, cat. no. F14217) or oxidative stress (DCF; Invitrogen, cat. no. C400). After washing, cells were imaged by Leica TCS SP8 confocal microscope (×60/1.4 NA oil objective). For calcium imaging, neurons and astrocytes were stimulated with KCl (Sigma–Aldrich, cat. no. P3911-25G) and glutamate (Sigma–Aldrich, cat. no. 49621-250 G), respectively. Fluorescence signals were quantified using Fiji following background subtraction and normalization to baseline. Detailed acquisition settings and analysis workflows are provided in Supplementary Methods.

### Phagocytosis assay

Microglia or tricultures were incubated with Alexa Fluor 488-labeled latex beads (Sigma‒Aldrich, cat. no. L1030) for 3 h at 37 °C; beads were pre-opsonized in fetal bovine serum (FBS, Thermo Scientific, cat. no. A5256801) for 1 h at 37 °C. Cells were washed with PBS (Thermo Scientific, cat. no. 14190169) and fixed with 4% paraformaldehyde for immunostaining. For flow cytometry, samples were dissociated with Accutase (STEMCELL Technologies, cat. no. 07920), incubated with Zombie NIR viability dye (1:1,000, BioLegend, cat. no. 423105) in PBS, washed in PBS with 2% FBS and analyzed on an Acea NovoCyte flow cytometer (Agilent Technologies) using FlowJo software (BD Biosciences; RRID:SCR_008520).

### Cytokine measurements

Cytokine levels in human iPSC-derived microglia were quantified using LEGENDplex bead-based immunoassays (BioLegend GmbH). IL-1β, IL-4, IL-6, TNF, IFN-γ, MCP-1 and IL-8 in cell supernatants were measured with the Human Essential Immune Response Panel (BioLegend GmbH, cat. no. 740930) and a Sony ID7000 spectral cell analyzer (Sony Biotechnology). Data (>300 beads per sample) were analyzed with LEGENDplex software v9.0 (BioLegend, https://www.biolegend.com/en-us/legendplex).

Cytokine levels in mouse brains were measured using Mouse TNF DuoSet ELISA (R&D Systems, cat. no. DY410) and Mouse IL-1β/Il-1F2 DuoSet ELISA (R&D Systems, cat. no. DY201). Whole brains from 6- and 15-month-old mice were rinsed with PBS and homogenized in ice-cold buffer (50 mM Tris-HCl, pH 7.4, 150 mM NaCl, 1% Triton X-100, 1 mM EDTA and protease/phosphatase inhibitors (Pierce, Thermo Scientific, cat. no. A32959) using 2 mm stainless steel beads. The lysates were centrifuged at 14,000*g* for 20 min, and the supernatants were collected for analysis. The absorbance was measured at 450 nm with wavelength correction at 540 nm using a PerkinElmer Envision 2105 plate reader. Cytokine concentrations were normalized to the total protein content determined by BCA assay (Pierce, Thermo Scientific, cat. no. 23225).

### Quantitative PCR with reverse transcription

mRNA was extracted from iPSC-derived neurons, astrocytes and microglia using the RNeasy Mini Kit (Qiagen, cat. no. 74106). cDNA was synthesized with the QuantiTect Reverse Transcription Kit (Qiagen, cat. no. 205313). Quantitative PCR was performed with SYBR Green PCR Master Mix (Qiagen, cat. no. 204145) on a Viia 7 Real-Time PCR System (Applied Biosystems). Gene expression was normalized to the housekeeping genes *RPLP0* or *ACTB*, and relative expression levels were calculated using the 2^−ΔΔCT^ method. Primer sequences are provided in Supplementary Table 2.

### Mitochondrial isolation

Mitochondrial isolation was performed with the QIAGEN Qproteome Mitochondria Isolation Kit (Qiagen, cat. no. 37612) with modifications. Pelleted cells (10 × 10^6^) were incubated on ice for 10 min in 1 ml of lysis buffer with protease inhibitor. The suspension was centrifuged at 1,000*g* for 10 min at 4 °C. Half of the supernatant was retained as the total cellular fraction, and the remainder was subjected to three additional centrifugation rounds (1,000*g*, 10 min, 4 °C) to obtain the cytosolic fraction. The pellet was resuspended in 1.5 ml of disruption buffer with protease inhibitors and mechanically disrupted by passing through a 26-gauge needle ten times on ice. After a first centrifugation (1,000*g*, 10 min, 4 °C), the pellet was discarded, and the supernatant was further centrifuged at 14,000*g* for 30 min at 4 °C. The final mitochondrial pellet was resuspended in 50 μl of storage buffer with protease inhibitors. Protein concentration was measured with the Pierce BCA Protein Assay Kit; 20 µg was used for immunoblotting and 30 µg for protein aggregation assays.

### Western blot

Protein lysates from cells and mouse brain tissue were prepared using detergent-based extraction buffers containing protease and phosphatase inhibitors. Protein concentrations were determined by BCA assay, and equal amounts of protein were resolved by SDS–PAGE (Surepage, GenScript, cat. no. M00654) and transferred to PVDF membranes (Thermo Scientific, cat. no. 10617354). Membranes were blocked and incubated with primary antibodies overnight, followed by HRP-conjugated secondary antibodies. Proteins were visualized using chemiluminescence and imaged with digital detection systems. Band intensities were quantified using ImageJ and normalized to loading controls. Antibodies are listed in Supplementary Table 1, and detailed buffer compositions and electrophoresis conditions are provided in Supplementary Methods.

### Protein aggregation measurement

Protein aggregation was assessed using the PROTEOSTAT Protein Aggregation Assay Kit (Enzo Life Sciences, cat. no. ENZ-51023-KP050) with modifications. The detection dye was diluted 1:2 in 1× assay buffer, and 2 µl was added per well of a black 96-well plate with clear bottom. Thirty micrograms of protein per sample were diluted to 100 µl in assay buffer, incubated 15 min at room temperature in the dark, and fluorescence was measured (550 nm excitation; 600 nm emission) using a PerkinElmer EnVision 2105 plate reader. For imaging-based detection, the PROTEOSTAT Aggresome Detection Kit (Enzo Life Sciences, cat. no. ENZ-51035-KIT) was used. Fixed cells were permeabilized with 0.1% Triton X-100 in PBS, incubated with the detection reagent for 30 min in the dark, counterstained with DAPI, and imaged on a Leica TCS SP8 confocal microscope (×60/1.4 NA oil objective).

### Bulk RNA-seq and data analysis

Total RNA from iPSC-derived neurons, astrocytes and microglia (three biological replicates per condition) treated with or without CDDO-Me was subjected to poly(A)-selected, strand-specific bulk RNA-seq (paired-end, 150 bp; Illumina NovaSeq). Differential gene expression analysis was performed using DESeq2 (version 1.44.0, RRID: SCR_015687), applying a fold-change (FC) cutoff >2 and Benjamini–Hochberg false discovery rate (FDR) < 0.05. Functional enrichment analysis of DEGs was conducted using gene ontology and pathway analysis tools, as described in Supplementary Methods. Overlap between cell-type-specific gene sets and published human microglial signatures was assessed using Fisher’s exact test. GSEA, (version v4.3.2, https://www.gsea-msigdb.org; RRID: SCR_003199) was performed using the Molecular Signatures database (MSigDB, version v7.5.1, RRID: SCR_016863). To evaluate enrichment of senescence-, SASP- and cell cycle-related pathways^25–28,85,77^, genes were ranked based on differential expression between treated and control samples, and significance was determined using normalized enrichment score (NES) and FDR correction. The original gene set files, RNA-seq read count input, sample label mapping, and full GSEA output reports for all three cell types have been deposited in Zenodo (10.5281/zenodo.20392968) to ensure complete traceability of the analysis. Full parameters and gene set details are provided in Supplementary Methods.

### Cell cycle analysis by flow cytometry

iPSC-derived cells were dissociated using Accutase, resuspended in PBS and stained with Hoechst 33342 (2.5 µg ml^−1^; Sigma–Aldrich, cat. no. 14533) for 30 min at 37 °C. After washing, cells were incubated with Zombie NIR viability dye (1:1,000 dilution in PBS). The cells were then washed and resuspended in PBS containing 2% FBS. Flow cytometry was performed on a Sony ID7000 spectral cell analyzer with a maximum event rate of 200 events per second. The data were analyzed in FlowJo software using the Watson (Pragmatic) model. The G1, S and G2/M phases were distinguished based on the Hoechst signal intensity. Model fitting was visualized by overlaying the model sum and the actual signal distribution. Peak 1 was set to ‘n’ and Peak 2 was constrained to match the coefficient of variation of the G1 peak. The final model selection was based on achieving a low root mean square deviation value.

### SA-β-gal staining

SA-β-gal staining was performed using the Senescence β-Galactosidase Staining Kit (Cell Signaling Technology, cat. no. 9860). Cells were cultured on Matrigel-coated coverslips and fixed with the provided fixation solution. For mouse tissue, OCT-embedded sections were cryosectioned, mounted on Epredia SuperFrost Plus slides (Thermo Scientific, cat. no. 10149870), refixed and stained following the manufacturer’s instructions. Images were acquired using a Leica DM750 brightfield microscope (×40; 0.4 NA) for cells or an EVOS M7000 Imaging System (×20; 0.4 NA; Thermo Scientific) for tissue sections.

### NAD consumption assay

NAD consumption was assessed with the fluorescent NAD analog nicotinamide 1,N^6^-ethenoadenine dinucleotide (Sigma–Aldrich, cat. no. N2630). iPSC-derived microglia (1 × 10^5^ per condition) were detached with Accutase and resuspended in 100 μl PBS. Eighty micromolar of the analog (340 nm excitation; 460 nm emission) was added, and fluorescence was recorded every minute for 1 h at 37 °C using a SpectraMax M2 reader (Molecular Devices). NADase activity was calculated from the linear slope and normalized to total protein content. Protein concentrations were determined with a Pierce BCA protein assay kit.

### Nuclear senescence scoring

iPSC-derived monocultures and tricultures were stained with DAPI and immunolabeled for MAP2, IBA1 and GFAP. Images were acquired using a Leica TCS SP8 confocal microscope equipped with a ×40, 1.4 NA oil immersion objective. Quantitative senescence scoring was performed as previously described^51^. Senescence prediction was based on nuclear morphology. Nuclei were segmented from DAPI images using a U-Net architecture, and morphometric features (area, convexity and aspect ratio) were extracted. Segmented nuclei were converted into two-channel masks and analyzed using an ensemble of deep neural networks to generate senescence probability scores. The models were trained originally on fibroblasts and validated across several cell types^51^. For triculture experiments, images were split into individual channels (IBA1, S100β and MAP2). A minimum integrated intensity threshold was applied to each channel to identify marker-positive cells. Objects exceeding the threshold were classified according to the channel with the highest integrated density signal. Analyses were performed using Python 3.9.18 (RRID:SCR_008394), TensorFlow GPU 2.15.0 (RRID:SCR_016345), Keras 2.15.0 (RRID:SCR_026159), NumPy 1.23.5 (RRID:SCR_008633) and Pandas 1.2.4 (RRID:SCR_018214).

### Lipidomic and metabolomic profiling of iPSC-derived microglia

Lipidomic analysis was conducted by Lipotype GmbH using high-resolution shotgun mass spectrometry on an Orbitrap platform with class-specific internal standards. Lipid species were identified based on mass spectrometry (MS) and tandem MS fragmentation patterns and quantified relative to internal standards. For metabolomics, polar metabolites were extracted using methanol/acetonitrile-based solvents, separated by hydrophilic interaction liquid chromatography, and analyzed by high-resolution Orbitrap mass spectrometry operated in full-scan polarity-switching mode. Metabolites were annotated based on accurate mass and retention time matching and quantified from peak areas. Statistical and pathway enrichment analyses were performed using established metabolomics workflows. Detailed extraction procedures, chromatographic gradients, mass spectrometry acquisition parameters, internal standards and data processing pipelines are provided in Supplementary Methods.

### scRNA-seq and analysis

Single-cell suspensions from iPSC-derived tricultures were processed using the 10x Genomics Chromium Next GEM Single Cell 3′ v3.1 kit (10x Genomics, cat. no. 1000128). Libraries were sequenced on an Illumina NovaSeq 6000 (SP Flow Cell, Illumina) paired-end to a depth of ~300 million reads per library (GENEWIZ GmbH). After Cell Ranger processing, a total of 7,160 high-quality cells were retained for analysis. Analyses were performed in Seurat (Seurat version 4.1.0, RRID: SCR_007322; R version 4.3.3, RRID: SCR_001905). Low-quality cells were excluded based on UMI count (<2,500), detected genes (<500) and mitochondrial transcript fraction (>20%). Doublets were removed using DoubletFinder (DoubletFinder version 3, RRID: SCR_018771). Data were normalized using SCTransform (version 0.4.1, RRID: SCR_022146), regressing out cell cycle and mitochondrial effects, and integrated using Harmony (version 1.2.0, RRID:SCR_022206). Clustering was performed using a shared nearest-neighbor graph based on the first 40 principal components, followed by UMAP visualization. Cluster identities were assigned using conserved marker genes. Differential expression within each cell type was assessed using the MAST test with Bonferroni correction. GSEA and pathway analysis were conducted on DEGs using standard functional enrichment tools. Cell–cell communication was analyzed using CellChat (version 2.1.0, RRID: SCR_021946). Additional details are provided in Supplementary Methods.

### Autophagic flux analysis

Autophagic flux in iPSC-derived microglia was assessed by treatment with 100 nM bafilomycin A1 (Sigma–Aldrich, cat. no. B1793) for 4 h before protein extraction. LC3B levels were analyzed by Western blot, and the LC3-II band intensity was quantified using ImageJ. Flux was calculated as the ratio of LC3-II in bafilomycin-treated cells versus untreated cells. Ratios were compared between control and CDDO-Me-treated conditions to evaluate autophagosome accumulation.

### α-synuclein preformed fibril treatment

Recombinant human α-synuclein was expressed, purified and assembled into PFFs as described previously^81^, with fibril formation validated by biochemical and ultrastructural assays (details in Supplementary Methods). Before use, PFFs were sonicated to generate short fibrillar species and added to iPSC-derived microglia at a final concentration of 1 µg ml^−1^. Where indicated, cells were cotreated with the indicated compounds. α-synuclein uptake and intracellular processing were assessed by immunofluorescence and live-cell imaging at defined timepoints. Particle quantification was performed using automated object detection and tracking using CellProfiler (version 4.2.6, RRID:SCR_007358).

### Statistics and reproducibility

Statistical analyses were performed using GraphPad Prism (version 10.2.3, RRID: SCR_002798). No statistical methods were used to predetermine sample sizes; sample sizes were comparable to those reported in previous iPSC-based studies. No formal randomization was performed. Cell cultures were assigned to experimental conditions based on availability and processed in parallel to minimize batch effects. Animals were grouped by genotype and age. Analyses were conducted blinded when feasible (for example, omics and automated image analyses using coded samples) but were otherwise unblinded. Samples were excluded if identified as statistical outliers or in cases of predefined technical failure. Data were assumed to follow a normal distribution but were not formally tested. Two-group comparisons were conducted using unpaired two-tailed Student’s *t*-tests, and multiple-group comparisons were analyzed by analysis of variance (ANOVA) with appropriate multiple-comparison corrections, as specified in the figure legends. Data are presented as mean ± s.e.m. from at least two independent differentiations with technical replicates.

### Reporting summary

Further information on research design is available in the Nature Portfolio Reporting Summary linked to this article.

## Online content

Any methods, additional references, Nature Portfolio reporting summaries, source data, extended data, supplementary information, acknowledgements, peer review information; details of author contributions and competing interests; and statements of data and code availability are available at 10.1038/s41593-026-02320-1.

## Supplementary information

Supplementary Information Supplementary Figs. 1–16, Methods, Tables 1–2 and Unprocessed blots.

Reporting Summary

Supplementary Data 1 Differentially expressed genes bulk RNA-seq.

Supplementary Data 2 Reactome analysis bulk RNA-seq.

Supplementary Data 3 Metabolomic analysis results.

Supplementary Data 4 Differentially expressed genes scRNA-seq.

Supplementary Data 5 VennPlot monocultures versus triculture.

Supplementary Data 6 Reactome analysis scRNA-seq.

Supplementary Data 7 Statistical source data for Supplementary Figures.

## Source data

Source Data Fig. 1 Statistical source data.

Source Data Fig. 2 Statistical source data.

Source Data Fig. 3 Statistical source data.

Source Data Fig. 4 Statistical source data.

Source Data Fig. 5 Statistical source data.

Source Data Fig. 6 Statistical source data.

Source Data Figs. 1 and 5 Unprocessed western blots.

## Data Availability

All data supporting the findings of this study are available within the article and its supplementary information files. Bulk and scRNA-seq datasets generated in this study have been deposited in the Gene Expression Omnibus (GEO) under accession numbers GSE274283 and GSE274289. Detailed experimental protocols are available on protocols.io at 10.17504/protocols.io.8epv5kbm4v1b/v1. Source data for all main and supplementary figures are provided in the accompanying files Source Data. The full collection of datasets, code, protocols, and key lab materials used and generated in this study are listed in a Key Resources Table alongside their persistent identifiers in Zenodo at 10.5281/zenodo.20507096 (ref. ^86^). An earlier version of this paper was posted to bioRxiv on 3 September 2024 at 10.1101/2024.09.03.610925. Previously published datasets used for comparative analyses are detailed in Methods with appropriate references. Source data are provided with this paper.

## References

[1] Zhao, Q. et al. A mitochondrial specific stress response in mammalian cells. *EMBO J.* **21**, 4411–4419 (2002). 12198143 10.1093/emboj/cdf445 PMC126185

[2] Shpilka, T. & Haynes, C. M. The mitochondrial UPR: mechanisms, physiological functions and implications in ageing. *Nat. Rev. Mol. Cell Biol.* **19**, 109–120 (2018). 29165426 10.1038/nrm.2017.110

[3] Houtkooper, R. H. et al. Mitonuclear protein imbalance as a conserved longevity mechanism. *Nature* **497**, 451–457 (2013). 23698443 10.1038/nature12188 PMC3663447

[4] Durieux, J., Wolff, S. & Dillin, A. The cell-non-autonomous nature of electron transport chain-mediated longevity. *Cell* **144**, 79–91 (2011). 21215371 10.1016/j.cell.2010.12.016 PMC3062502

[5] Begcevic, I. et al. Semiquantitative proteomic analysis of human hippocampal tissues from Alzheimer’s disease and age-matched control brains. *Clin. Proteomics* **10**, 5 (2013). 23635041 10.1186/1559-0275-10-5 PMC3648498

[6] Brunetti, D. et al. Targeting multiple mitochondrial processes by a metabolic modulator prevents sarcopenia and cognitive decline in SAMP8 mice. *Front. Pharmacol.* **11**, 1171 (2020). 32848778 10.3389/fphar.2020.01171 PMC7411305

[7] Cooper, J. F. et al. Activation of the mitochondrial unfolded protein response promotes longevity and dopamine neuron survival in Parkinson’s disease models. *Sci. Rep.* **7**, 16441 (2017). 29180793 10.1038/s41598-017-16637-2 PMC5703891

[8] Perez, M. J. et al. Loss of function of the mitochondrial peptidase PITRM1 induces proteotoxic stress and Alzheimer’s disease-like pathology in human cerebral organoids. *Mol. Psychiatry* **26**, 5733–5750 (2021). 32632204 10.1038/s41380-020-0807-4 PMC8758476

[9] Sorrentino, V. et al. Enhancing mitochondrial proteostasis reduces amyloid-beta proteotoxicity. *Nature* **552**, 187–193 (2017). 29211722 10.1038/nature25143 PMC5730497

[10] Lin, Y. F. et al. Maintenance and propagation of a deleterious mitochondrial genome by the mitochondrial unfolded protein response. *Nature* **533**, 416–419 (2016). 27135930 10.1038/nature17989 PMC4873342

[11] Martinez, B. A. et al. Dysregulation of the mitochondrial unfolded protein response induces non-apoptotic dopaminergic neurodegeneration in *C. elegans* models of Parkinson’s disease. *J. Neurosci.* **37**, 11085–11100 (2017). 29030433 10.1523/JNEUROSCI.1294-17.2017 PMC5688521

[12] Jeong, D. E. et al. Mitochondrial chaperone HSP-60 regulates anti-bacterial immunity via p38 MAP kinase signaling. *EMBO J.* **36**, 1046–1065 (2017). 28283579 10.15252/embj.201694781 PMC5391144

[13] Pellegrino, M. W. et al. Mitochondrial UPR-regulated innate immunity provides resistance to pathogen infection. *Nature* **516**, 414–417 (2014). 25274306 10.1038/nature13818 PMC4270954

[14] Bar-Ziv et al. Glial-derived mitochondrial signals affect neuronal proteostasis and aging. *Sci. Adv.* **9**, eadi1411 (2023). 37831769 10.1126/sciadv.adi1411 PMC10575585

[15] Berendzen, K. M. et al. Neuroendocrine coordination of mitochondrial stress signaling and proteostasis. *Cell* **166**, 1553–1563 (2016). 27610575 10.1016/j.cell.2016.08.042 PMC5922979

[16] Shao, L. W., Niu, R. & Liu, Y. Neuropeptide signals cell non-autonomous mitochondrial unfolded protein response. *Cell Res.* **26**, 1182–1196 (2016). 27767096 10.1038/cr.2016.118 PMC5099867

[17] Zhang, Q. et al. The mitochondrial unfolded protein response is mediated cell-non-autonomously by retromer-dependent Wnt signaling. *Cell* **174**, 870–883 (2018). 30057120 10.1016/j.cell.2018.06.029 PMC6086732

[18] Lee, J. et al. Inhibition of mitochondrial LonP1 protease by allosteric blockade of ATP -binding and -hydrolysis via CDDO and its derivatives. *J. Biol. Chem.* **298**, 101719 (2022). 35151690 10.1016/j.jbc.2022.101719 PMC8921294

[19] Fiorese, C. J. et al. The transcription factor ATF5 mediates a mammalian mitochondrial UPR. *Curr. Biol.* **26**, 2037–2043 (2016). 27426517 10.1016/j.cub.2016.06.002 PMC4980197

[20] Katiyar, A. et al. HSF1 is required for induction of mitochondrial chaperones during the mitochondrial unfolded protein response. *FEBS Open Bio* **10**, 1135–1148 (2020). 32302062 10.1002/2211-5463.12863 PMC7262932

[21] Novoa, I. et al. Stress-induced gene expression requires programmed recovery from translational repression. *EMBO J.* **22**, 1180–1187 (2003). 12606582 10.1093/emboj/cdg112 PMC150345

[22] Guan, B. J. et al. A unique ISR program determines cellular responses to chronic stress. *Mol. Cell* **68**, 885–900 (2017). 29220654 10.1016/j.molcel.2017.11.007 PMC5730339

[23] Avelar, R. A. et al. Integrated stress response plasticity governs normal cell adaptation to chronic stress via the PP2A-TFE3-ATF4 pathway. *Cell Death Differ.* **31**, 1761–1775 (2024). 39349971 10.1038/s41418-024-01378-3 PMC11618521

[24] Flury, A. et al. A neurodegenerative cellular stress response linked to dark microglia and toxic lipid secretion. *Neuron* **113**, 554–571 (2025). 39719704 10.1016/j.neuron.2024.11.018 PMC12481204

[25] Casella, G. et al. Transcriptome signature of cellular senescence. *Nucleic Acids Res.* **47**, 11476 (2019). 31612919 10.1093/nar/gkz879 PMC6868356

[26] Fridman, A. L. & Tainsky, M. A. Critical pathways in cellular senescence and immortalization revealed by gene expression profiling. *Oncogene* **27**, 5975–5987 (2008). 18711403 10.1038/onc.2008.213 PMC3843241

[27] Kamminga, L. M. & de Haan, G. Cellular memory and hematopoietic stem cell aging. *Stem Cells* **24**, 1143–1149 (2006). 16456126 10.1634/stemcells.2005-0345

[28] Hernandez-Segura, A. et al. Unmasking transcriptional heterogeneity in senescent cells. *Curr. Biol.* **27**, 2652–2660 (2017). 28844647 10.1016/j.cub.2017.07.033 PMC5788810

[29] Reimann, M., Lee, S. & Schmitt, C. A. Cellular senescence: neither irreversible nor reversible. *J. Exp. Med.* **221**, e20232136 (2024). 38385946 10.1084/jem.20232136 PMC10883852

[30] Yegorov, Y. E., Akimov, S. S., Hass, R., Zelenin, A. V. & Prudovsky, I. A. Endogenous beta-galactosidase activity in continuously nonproliferating cells. *Exp. Cell. Res.* **243**, 207–211 (1998). 9716464 10.1006/excr.1998.4169

[31] Gorgoulis, V. et al. Cellular senescence: defining a path forward. *Cell* **179**, 813–827 (2019). 31675495 10.1016/j.cell.2019.10.005

[32] Covarrubias, A. J. et al. Senescent cells promote tissue NAD^+^ decline during ageing via the activation of CD38^+^ macrophages. *Nat. Metab.* **2**, 1265–1283 (2020). 33199924 10.1038/s42255-020-00305-3 PMC7908681

[33] Gibson, B. A. & Kraus, W. L. New insights into the molecular and cellular functions of poly(ADP-ribose) and PARPs. *Nat. Rev. Mol. Cell Biol.* **13**, 411–424 (2012). 22713970 10.1038/nrm3376

[34] Nacarelli, T. et al. NAD^+^ metabolism governs the proinflammatory senescence-associated secretome. *Nat. Cell Biol.* **21**, 397–407 (2019). 30778219 10.1038/s41556-019-0287-4 PMC6448588

[35] Munch, C. & Harper, J. W. Mitochondrial unfolded protein response controls matrix pre-RNA processing and translation. *Nature* **534**, 710–713 (2016). 27350246 10.1038/nature18302 PMC4939261

[36] Matt, S. M., Lawson, M. A. & Johnson, R. W. Aging and peripheral lipopolysaccharide can modulate epigenetic regulators and decrease IL-1beta promoter DNA methylation in microglia. *Neurobiol. Aging* **47**, 1–9 (2016). 27500965 10.1016/j.neurobiolaging.2016.07.006 PMC5075520

[37] Nikodemova, M., Small, A. L., Kimyon, R. S. & Watters, J. J. Age-dependent differences in microglial responses to systemic inflammation are evident as early as middle age. *Physiol. Genomics* **48**, 336–344 (2016). 26884461 10.1152/physiolgenomics.00129.2015 PMC4855213

[38] Martins-Ferreira, R. et al. The Human Microglia Atlas (HuMicA) unravels changes in disease-associated microglia subsets across neurodegenerative conditions. *Nat. Commun.* **16**, 739 (2025). 39820004 10.1038/s41467-025-56124-1 PMC11739505

[39] Gerrits, E. et al. Distinct amyloid-beta and tau-associated microglia profiles in Alzheimer’s disease. *Acta Neuropathol.* **141**, 681–696 (2021). 33609158 10.1007/s00401-021-02263-w PMC8043951

[40] Sun, N. et al. Human microglial state dynamics in Alzheimer’s disease progression. *Cell* **186**, 4386–4403 (2023). 37774678 10.1016/j.cell.2023.08.037 PMC10644954

[41] Olah, M. et al. Single cell RNA sequencing of human microglia uncovers a subset associated with Alzheimer’s disease. *Nat. Commun.* **11**, 6129 (2020). 33257666 10.1038/s41467-020-19737-2 PMC7704703

[42] Silvin, A. et al. Dual ontogeny of disease-associated microglia and disease inflammatory macrophages in aging and neurodegeneration. *Immunity* **55**, 1448–1465 (2022). 35931085 10.1016/j.immuni.2022.07.004

[43] Stahl, A. et al. Isolation and identification of a novel mitochondrial metalloprotease (PreP) that degrades targeting presequences in plants. *J. Biol. Chem.* **277**, 41931–41939 (2002). 12138166 10.1074/jbc.M205500200

[44] Poveda-Huertes, D. et al. An early mtUPR: redistribution of the nuclear transcription factor Rox1 to mitochondria protects against intramitochondrial proteotoxic aggregates. *Mol. Cell* **77**, 180–188 (2020). 31630969 10.1016/j.molcel.2019.09.026 PMC6941230

[45] Brunetti, D. et al. Defective PITRM1 mitochondrial peptidase is associated with Abeta amyloidotic neurodegeneration. *EMBO Mol. Med.* **8**, 176–190 (2016). 26697887 10.15252/emmm.201505894 PMC4772954

[46] Langer, Y. et al. Mitochondrial PITRM1 peptidase loss-of-function in childhood cerebellar atrophy. *J. Med. Genet.* **55**, 599–606 (2018). 29764912 10.1136/jmedgenet-2018-105330

[47] Alikhani, N. et al. Decreased proteolytic activity of the mitochondrial amyloid-beta degrading enzyme, PreP peptidasome, in Alzheimer’s disease brain mitochondria. *J. Alzheimers Dis.* **27**, 75–87 (2011). 21750375 10.3233/JAD-2011-101716 PMC3381900

[48] Falkevall, A. et al. Degradation of the amyloid beta-protein by the novel mitochondrial peptidasome, PreP. *J. Biol. Chem.* **281**, 29096–29104 (2006). 16849325 10.1074/jbc.M602532200

[49] Young, A. R., Narita, M. & Narita, M. Cell senescence as both a dynamic and a static phenotype. *Methods Mol. Biol.* **965**, 1–13 (2013). 23296648 10.1007/978-1-62703-239-1_1

[50] Basisty, N. et al. A proteomic atlas of senescence-associated secretomes for aging biomarker development. *PLoS Biol.* **18**, e3000599 (2020). 31945054 10.1371/journal.pbio.3000599 PMC6964821

[51] Heckenbach, I. et al. Nuclear morphology is a deep learning biomarker of cellular senescence. *Nat. Aging* **2**, 742–755 (2022). 37118134 10.1038/s43587-022-00263-3 PMC10154217

[52] Kim, H. E. et al. Lipid biosynthesis coordinates a mitochondrial-to-cytosolic stress response. *Cell* **166**, 1539–1552 (2016). 27610574 10.1016/j.cell.2016.08.027 PMC5922983

[53] Tighanimine, K. et al. A homoeostatic switch causing glycerol-3-phosphate and phosphoethanolamine accumulation triggers senescence by rewiring lipid metabolism. *Nat. Metab.* **6**, 323–342 (2024). 38409325 10.1038/s42255-023-00972-y PMC10896726

[54] Pang, Z. et al. Using MetaboAnalyst 5.0 for LC-HRMS spectra processing, multi-omics integration and covariate adjustment of global metabolomics data. *Nat. Protoc.* **17**, 1735–1761 (2022). 35715522 10.1038/s41596-022-00710-w

[55] Sutandy, F. X. R., Gossner, I., Tascher, G. & Munch, C. A cytosolic surveillance mechanism activates the mitochondrial UPR. *Nature* **618**, 849–854 (2023). 37286597 10.1038/s41586-023-06142-0 PMC10284689

[56] Jin, S. et al. Inference and analysis of cell-cell communication using CellChat. *Nat. Commun.* **12**, 1088 (2021). 33597522 10.1038/s41467-021-21246-9 PMC7889871

[57] De Schepper, S. et al. Perivascular cells induce microglial phagocytic states and synaptic engulfment via SPP1 in mouse models of Alzheimer’s disease. *Nat. Neurosci.* **26**, 406–415 (2023). 36747024 10.1038/s41593-023-01257-z PMC9991912

[58] Jin, M. et al. Type-I-interferon signaling drives microglial dysfunction and senescence in human iPSC models of Down syndrome and Alzheimer’s disease. *Cell Stem Cell* **29**, 1135–1153 (2022). 35803230 10.1016/j.stem.2022.06.007 PMC9345168

[59] van der Kant, R., Goldstein, L. S. B. & Ossenkoppele, R. Amyloid-beta-independent regulators of tau pathology in Alzheimer disease. *Nat. Rev. Neurosci.* **21**, 21–35 (2020). 31780819 10.1038/s41583-019-0240-3

[60] Gulisano, W. et al. Role of amyloid-beta and tau proteins in Alzheimer’s disease: confuting the amyloid cascade. *J. Alzheimers Dis.* **64**, S611–S631 (2018). 29865055 10.3233/JAD-179935 PMC8371153

[61] Hampel, H. et al. The amyloid-beta pathway in Alzheimer’s disease. *Mol. Psychiatry* **26**, 5481–5503 (2021). 34456336 10.1038/s41380-021-01249-0 PMC8758495

[62] Baker, B. M., Nargund, A. M., Sun, T. & Haynes, C. M. Protective coupling of mitochondrial function and protein synthesis via the eIF2alpha kinase GCN-2. *PLoS Genet.* **8**, e1002760 (2012). 22719267 10.1371/journal.pgen.1002760 PMC3375257

[63] Higuchi-Sanabria, R. et al. Divergent nodes of non-autonomous UPR(ER) signaling through serotonergic and dopaminergic neurons. *Cell Rep.* **33**, 108489 (2020). 33296657 10.1016/j.celrep.2020.108489 PMC8820220

[64] Ogrodnik, M. et al. Guidelines for minimal information on cellular senescence experimentation in vivo. *Cell* **187**, 4150–4175 (2024). 39121846 10.1016/j.cell.2024.05.059 PMC11790242

[65] Jurk, D. et al. Postmitotic neurons develop a p21-dependent senescence-like phenotype driven by a DNA damage response. *Aging Cell* **11**, 996–1004 (2012). 22882466 10.1111/j.1474-9726.2012.00870.x PMC3533793

[66] Piechota, M. et al. Is senescence-associated beta-galactosidase a marker of neuronal senescence?. *Oncotarget* **7**, 81099–81109 (2016). 27768595 10.18632/oncotarget.12752 PMC5348379

[67] Oikonomou, G. & Shaham, S. The glia of *Caenorhabditis elegans*. *Glia* **59**, 1253–1263 (2011). 21732423 10.1002/glia.21084 PMC3117073

[68] Bae, E. J. et al. TNF-alpha promotes alpha-synuclein propagation through stimulation of senescence-associated lysosomal exocytosis. *Exp. Mol. Med.* **54**, 788–800 (2022). 35790884 10.1038/s12276-022-00789-x PMC9352737

[69] Marschallinger, J. et al. Lipid-droplet-accumulating microglia represent a dysfunctional and proinflammatory state in the aging brain. *Nat. Neurosci.* **23**, 194–208 (2020). 31959936 10.1038/s41593-019-0566-1 PMC7595134

[70] Haney, M. S. et al. APOE4/4 is linked to damaging lipid droplets in Alzheimer’s disease microglia. *Nature* **628**, 154–161 (2024). 38480892 10.1038/s41586-024-07185-7 PMC10990924

[71] Baker, Z. N. et al. Triacylglycerol mobilization underpins mitochondrial stress recovery. *Nat. Cell Biol.* **27**, 298–308 (2025). 39779944 10.1038/s41556-024-01586-6 PMC11821527

[72] Labbe, K. et al. Specific activation of the integrated stress response uncovers regulation of central carbon metabolism and lipid droplet biogenesis. *Nat. Commun.* **15**, 8301 (2024). 39333061 10.1038/s41467-024-52538-5 PMC11436933

[73] Sharma, A. K., Khandelwal, R. & Wolfrum, C. Futile cycles: emerging utility from apparent futility. *Cell Metab.* **36**, 1184–1203 (2024). 38565147 10.1016/j.cmet.2024.03.008

[74] Sharma, A. K., Khandelwal, R. & Wolfrum, C. Futile lipid cycling: from biochemistry to physiology. *Nat. Metab.* **6**, 808–824 (2024). 38459186 10.1038/s42255-024-01003-0

[75] Byrns, C. N. et al. Senescent glia link mitochondrial dysfunction and lipid accumulation. *Nature* **630**, 475–483 (2024). 38839958 10.1038/s41586-024-07516-8 PMC11168935

[76] Chen, T. Y., Wang, F. Y., Lee, P. J., Hsu, A. L. & Ching, T. T. Mitochondrial S-adenosylmethionine deficiency induces mitochondrial unfolded protein response and extends lifespan in *Caenorhabditis elegans*. *Aging Cell* **23**, e14103 (2024). 38361361 10.1111/acel.14103 PMC11019128

[77] Saul, D. et al. A new gene set identifies senescent cells and predicts senescence-associated pathways across tissues. *Nat. Commun.* **13**, 4827 (2022). 35974106 10.1038/s41467-022-32552-1 PMC9381717

[78] Tang, X. et al. Amyloid-β precursor-like protein APLP1 is a novel p53 transcriptional target gene that augments neuroblastoma cell death upon genotoxic stress. *Oncogene* **26**, 7302–7312 (2007). 17533371 10.1038/sj.onc.1210542

[79] Schondorf, D. C. et al. The NAD^+^ precursor nicotinamide riboside rescues mitochondrial defects and neuronal loss in iPSC and fly models of Parkinson’s disease. *Cell Rep.* **23**, 2976–2988 (2018). 29874584 10.1016/j.celrep.2018.05.009

[80] Panagiotakopoulou, V. et al. Interferon-gamma signaling synergizes with LRRK2 in neurons and microglia derived from human induced pluripotent stem cells. *Nat. Commun.* **11**, 5163 (2020). 33057020 10.1038/s41467-020-18755-4 PMC7560616

[81] Baden, P. et al. Glucocerebrosidase is imported into mitochondria and preserves complex I integrity and energy metabolism. *Nat. Commun.* **14**, 1930 (2023). 37024507 10.1038/s41467-023-37454-4 PMC10079970

[82] Tcw, J. et al. An efficient platform for astrocyte differentiation from human induced pluripotent stem cells. *Stem Cell Rep.* **9**, 600–614 (2017). 10.1016/j.stemcr.2017.06.018 PMC5550034 28757165

[83] Haenseler, W. et al. A highly efficient human pluripotent stem cell microglia model displays a neuronal-co-culture-specific expression profile and inflammatory response. *Stem Cell Rep.* **8**, 1727–1742 (2017). 10.1016/j.stemcr.2017.05.017 PMC5470330 28591653

[84] Revah, O. et al. Maturation and circuit integration of transplanted human cortical organoids. *Nature* **610**, 319–326 (2022). 36224417 10.1038/s41586-022-05277-w PMC9556304

[85] Subramanian, A. et al. Gene set enrichment analysis: a knowledge-based approach for interpreting genome-wide expression profiles. *Proc. Natl Acad. Sci. USA* **102**, 15545–15550 (2005). 16199517 10.1073/pnas.0506580102 PMC1239896

[86] Deleidi, M. & Pérez-Jiménez, M. J. Key Resources Table _The mitochondrial unfolded protein response in human microglia disrupts neuronal-glial communication and promotes senescence. *Zenodo*10.5281/zenodo.20507096 (2026). 10.1038/s41593-026-02320-1 PMC13433254 42362883

