# Supplementary material for: The mitochondrial unfolded protein response in human microglia disrupts neuronal–glial communication and promotes senescence
Source: Nat Neurosci. 2026 Jun 26;29(8):1858–72. doi: 10.1038/s41593-026-02320-1 (PMC13433254; doi:10.1038/s41593-026-02320-1)

1B. Proteostat measurements in iPSC-derived lines treated with CDDO-Me

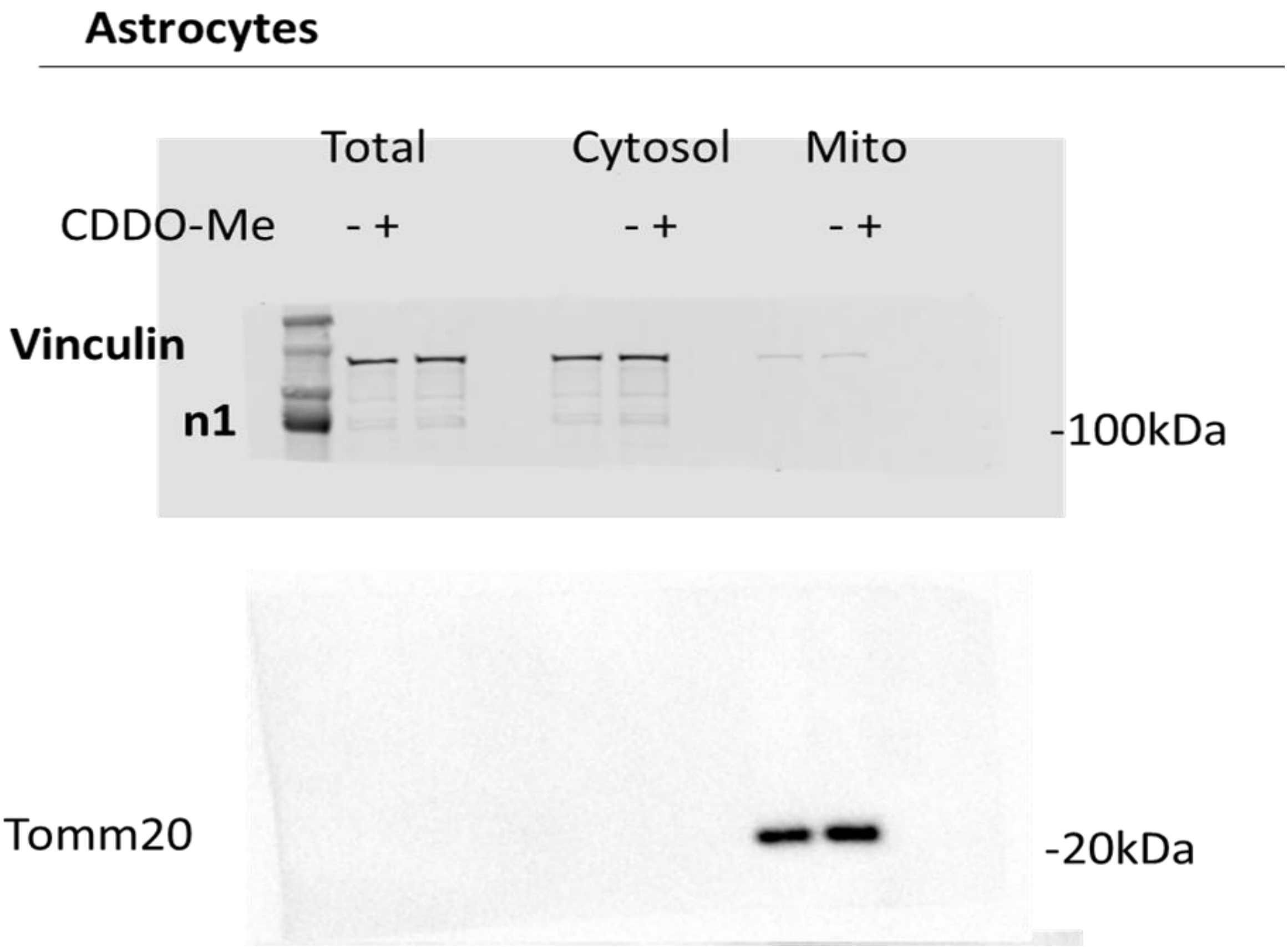

1B. Proteostat measurements in iPSC-derived lines treated with CDDO-Me

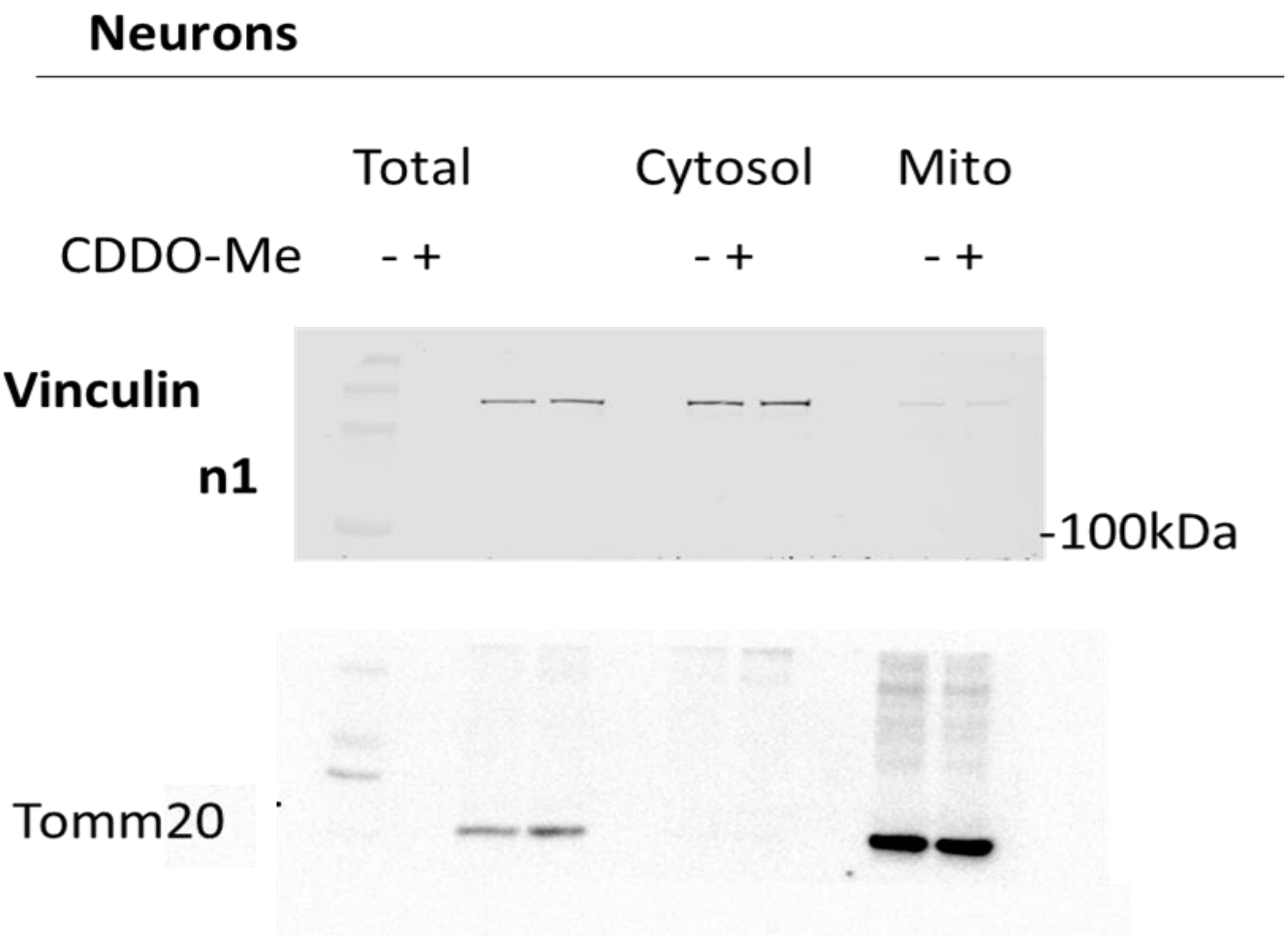

1B. Proteostat measurements in iPSC-derived lines treated with CDDO-Me

Microglia

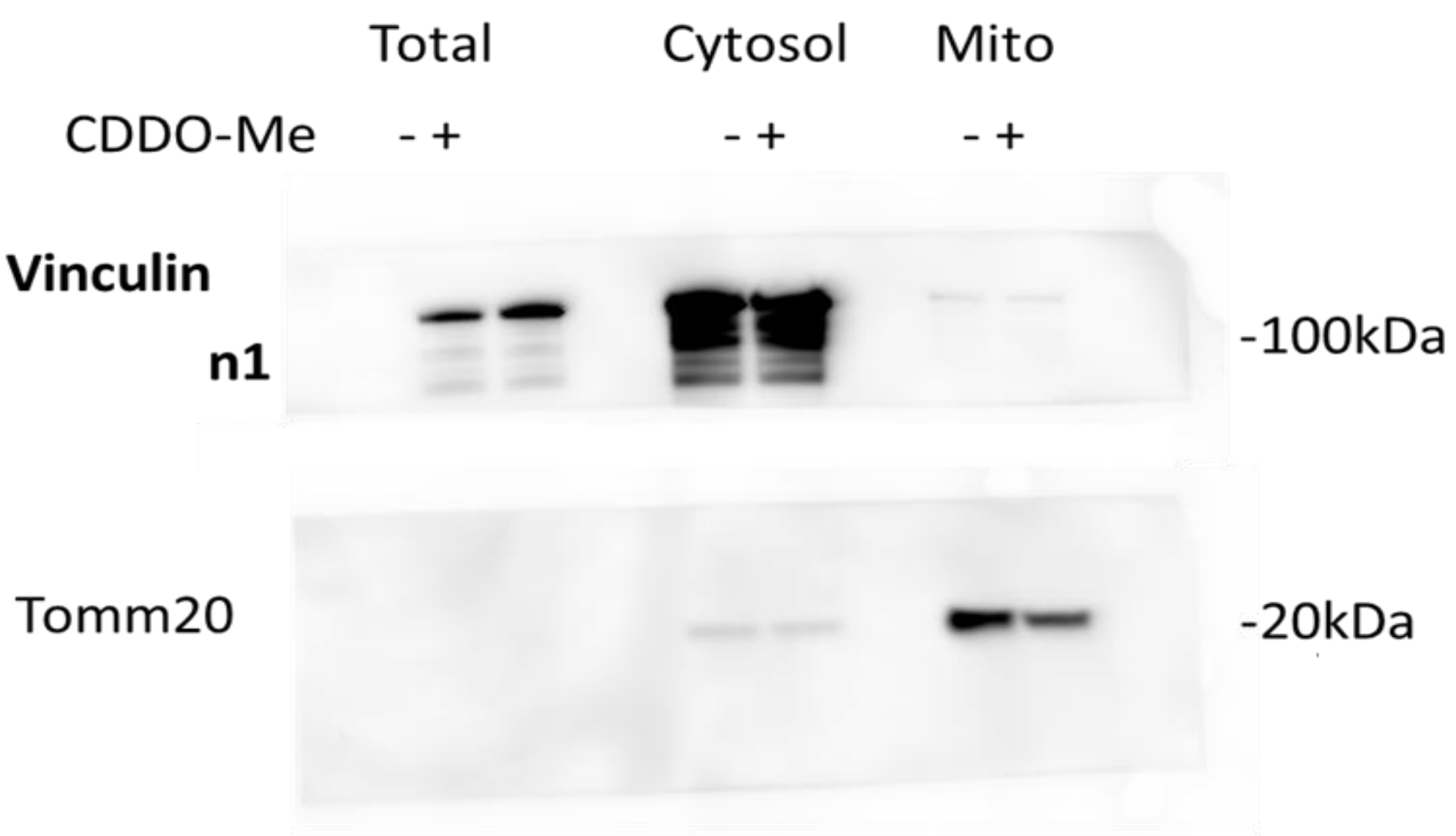

5H. Quantification autophagy flux

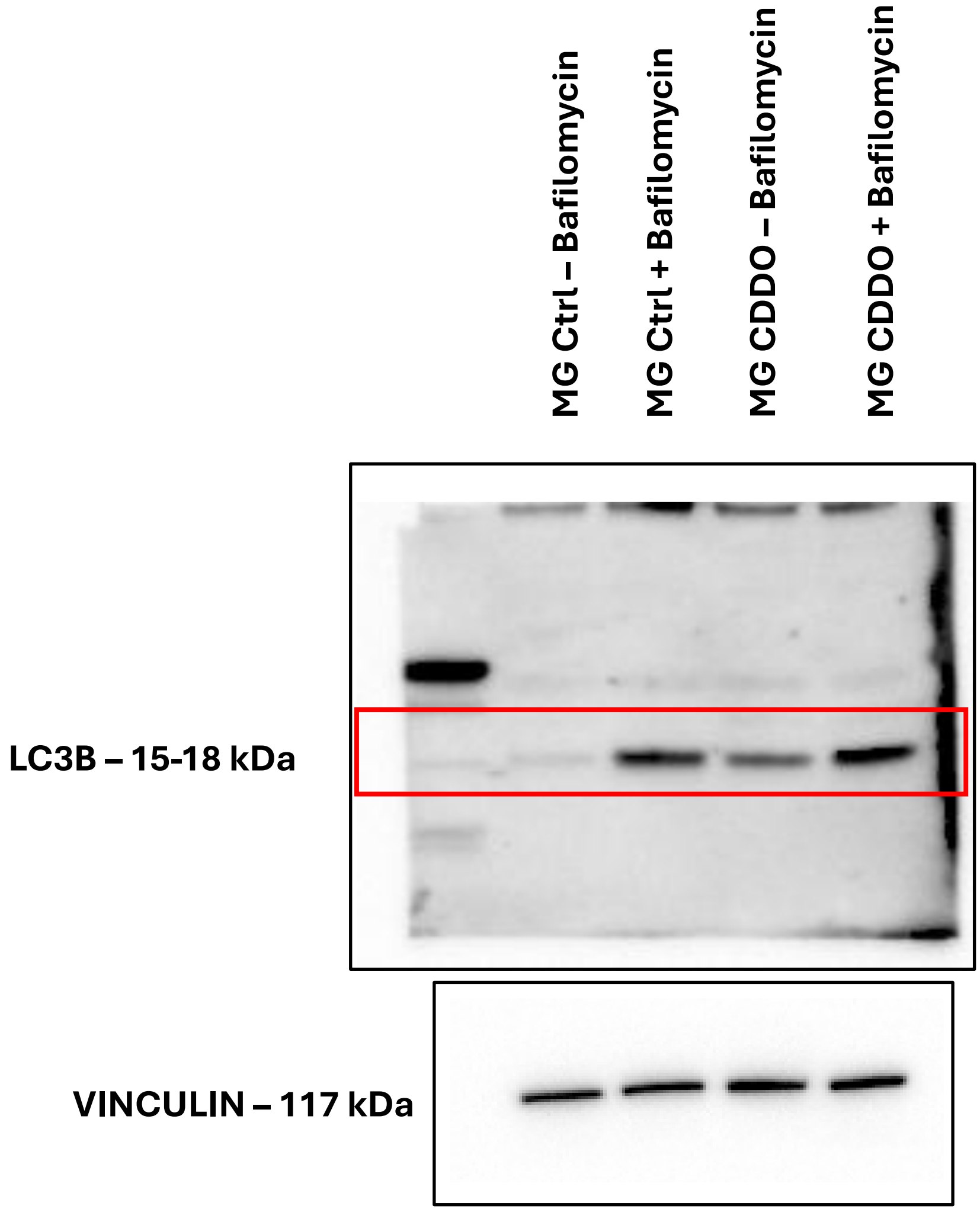

5H. Quantification autophagy flux

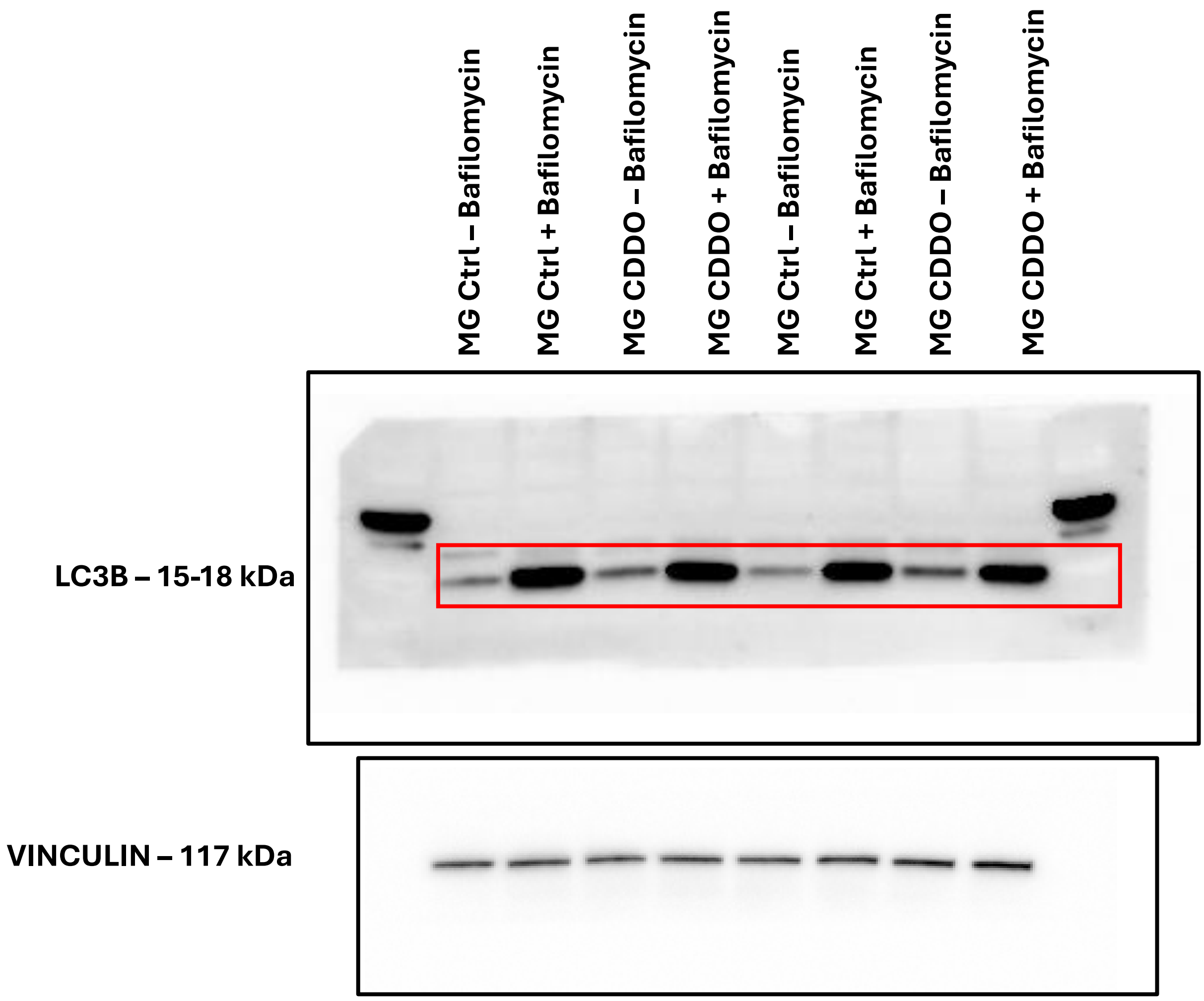

Supplement: Supplementary file 1 [file 41593_2026_2320_MOESM16_ESM.pdf]
