# Supplementary material for: The mitochondrial unfolded protein response in human microglia disrupts neuronal–glial communication and promotes senescence
Source: Nat Neurosci. 2026 Jun 26;29(8):1858–72. doi: 10.1038/s41593-026-02320-1 (PMC13433254; doi:10.1038/s41593-026-02320-1)
Supplement: Supplementary file 3 [file 41593_2026_2320_MOESM2_ESM.pdf]

nature portfolio

Corresponding author(s): Michela Deleidi, Maria Jose Perez

Last updated by author(s): Mar 6, 2026

Reporting Summary

Nature Portfolio wishes to improve the reproducibility of the work that we publish. This form provides structure for consistency and transparency in reporting. For further information on Nature Portfolio policies, see our Editorial Policies and the Editorial Policy Checklist.

Statistics

For all statistical analyses, confirm that the following items are present in the figure legend, table legend, main text, or Methods section.

n/a	Confirmed	
☐	☒	The exact sample size (*n*) for each experimental group/condition, given as a discrete number and unit of measurement
☐	☒	A statement on whether measurements were taken from distinct samples or whether the same sample was measured repeatedly
☐	☒	The statistical test(s) used AND whether they are one- or two-sided<br>*Only common tests should be described solely by name; describe more complex techniques in the Methods section.*
☒	☐	A description of all covariates tested
☐	☒	A description of any assumptions or corrections, such as tests of normality and adjustment for multiple comparisons
☐	☒	A full description of the statistical parameters including central tendency (e.g. means) or other basic estimates (e.g. regression coefficient) AND variation (e.g. standard deviation) or associated estimates of uncertainty (e.g. confidence intervals)
☐	☒	For null hypothesis testing, the test statistic (e.g. *F*, *t*, *r*) with confidence intervals, effect sizes, degrees of freedom and *P* value noted<br>*Give P values as exact values whenever suitable.*
☒	☐	For Bayesian analysis, information on the choice of priors and Markov chain Monte Carlo settings
☒	☐	For hierarchical and complex designs, identification of the appropriate level for tests and full reporting of outcomes
☒	☐	Estimates of effect sizes (e.g. Cohen's *d*, Pearson's *r*), indicating how they were calculated

Our web collection on statistics for biologists contains articles on many of the points above.

Software and code

Policy information about availability of computer code

Data collection

Images were acquired with a Leica TCS SP8 confocal microscope with a 60×/1.4 numerical-aperture oil-immersion objective (Leica), controlled with LAS X v3.7 software; with an EVOS M7000 Imaging System microscope with a 20×/0.4 numerical aperture (Thermo Fisher Scientific), using EVOS software v1.6; with a Leica DM750 bright-field microscope with a 40×/0.4 numerical-aperture objective (Leica), Leica Application Suite v4.13; or with an Evident APX100-HCU box-type microscope with a 20×/0.8 numerical-aperture objective (Evident, Japan), cellSens v4.2.Cytokine levels, cell cycle profiles, phagocytosis and Zombie NIR stainings were measured on a Sony ID7000™ spectral cell analyzer (Sony Biotechnology, USA), ID7000 software v1.8.Quantitative PCR was performed on a Viia 7 Real-Time PCR System (Applied Biosystems, CA, USA) running Viia 7 RUO v1.2.4.High-quality RNA samples were subjected to bulk RNA-seq at GENEWIZ GmbH (Leipzig, Germany). Sequencing was performed on an Illumina NovaSeq 6000 platform (Illumina, CA, USA) with S4 flow cells, and base calls were converted to FASTQ with bcl2fastq v2.20.A fluorescence plate reader (PerkinElmer Envision 2105; PerkinElmer, Waltham, MA, USA) running EnVision Manager v1.13, or a SpectraMax M2 Multi-Mode microplate reader (Molecular Devices) running SoftMax Pro v7.1, was used for fluorescence analysis.Western blots were visualized using an Odyssey DLx Imager (LI-COR), Image Studio v5.2; a Stella imaging system (Raytest), Control v1.7; or a ChemiDoc MP imaging system (Bio-Rad), Image Lab v6.1.LC–MS analyses were conducted with a Q Exactive Plus Orbitrap mass spectrometer equipped with an Ion Max source and a HESI II probe coupled to a Dionex UltiMate 3000 UHPLC system (Thermo Fisher Scientific). Data were acquired with Xcalibur software (Thermo Fisher Scientific) v4.1.10× Chromium libraries were sequenced on the Illumina NovaSeq 6000 platform (SP Flow Cell, Illumina) under the same run parameters as bulk RNA-seq.Lipidomic profiling was performed on the same Q Exactive Plus Orbitrap instrument, and data were analyzed with Compound Discoverer 3.3 (Thermo Fisher Scientific) with normalization against internal standards and total protein.All instruments were calibrated according to the manufacturers' recommendations, and no non-standard acquisition settings were applied.

Data analysis

Images were acquired with a Leica TCS SP8 confocal microscope with a 60×/1.4 numerical-aperture oil-immersion objective (Leica), controlled with LAS X v3.7 software; with an EVOS M7000 Imaging System microscope with a 20×/0.4 numerical aperture (Thermo Fisher Scientific),

using EVOS software v1.6; with a Leica DM750 bright-field microscope with a 40×/0.4 numerical-aperture objective (Leica), Leica Application Suite v4.13; or with an Evident APX100-HCU box-type microscope with a 20×/0.8 numerical-aperture objective (Evident, Japan), cellSens v4.2.Cytokine levels, cell cycle profiles, phagocytosis and Zombie NIR stainings were measured on a Sony ID7000™ spectral cell analyzer (Sony Biotechnology, USA), ID7000 software v1.8.Quantitative PCR was performed on a Viia 7 Real-Time PCR System (Applied Biosystems, CA, USA) running Viia 7 RUO v1.2.4.High-quality RNA samples were subjected to bulk RNA-seq at GENEWIZ GmbH (Leipzig, Germany). Sequencing was performed on an Illumina NovaSeq 6000 platform (Illumina, CA, USA) with S4 flow cells, and base calls were converted to FASTQ with bcl2fastq v2.20.A fluorescence plate reader (PerkinElmer Envision 2105; PerkinElmer, Waltham, MA, USA) running EnVision Manager v1.13, or a SpectraMax M2 Multi-Mode microplate reader (Molecular Devices) running SoftMax Pro v7.1, was used for fluorescence analysis.Western blots were visualized using an Odyssey DLx Imager (LI-COR), Image Studio v5.2; a Stella imaging system (Raytest), Control v1.7; or a ChemiDoc MP imaging system (Bio-Rad), Image Lab v6.1.LC–MS analyses were conducted with a Q Exactive Plus Orbitrap mass spectrometer equipped with an Ion Max source and a HESI II probe coupled to a Dionex UltiMate 3000 UHPLC system (Thermo Fisher Scientific). Data were acquired with Xcalibur software (Thermo Fisher Scientific) v4.1.10× Chromium libraries were sequenced on the Illumina NovaSeq 6000 platform (SP Flow Cell, Illumina) under the same run parameters as bulk RNA-seq.Lipidomic profiling was performed on the same Q Exactive Plus Orbitrap instrument, and data were analyzed with Compound Discoverer 3.3 (Thermo Fisher Scientific) with normalization against internal standards and total protein.All instruments were calibrated according to the manufacturers' recommendations, and no non-standard acquisition settings were applied.

For manuscripts utilizing custom algorithms or software that are central to the research but not yet described in published literature, software must be made available to editors and reviewers. We strongly encourage code deposition in a community repository (e.g. GitHub). See the Nature Portfolio guidelines for submitting code & software for further information.

Data

Policy information about availability of data

All manuscripts must include a data availability statement. This statement should provide the following information, where applicable:

- Accession codes, unique identifiers, or web links for publicly available datasets
- A description of any restrictions on data availability
- For clinical datasets or third party data, please ensure that the statement adheres to our policy

The main data supporting the findings of this study are available within the article and its Supplementary files. The transcriptomic data have been deposited into GEO under accession numbers: GSE274283 and GSE274289.

Research involving human participants, their data, or biological material

Policy information about studies with human participants or human data. See also policy information about sex, gender (identity/presentation), and sexual orientation and race, ethnicity and racism.

Reporting on sex and gender	Sex and gender were not considered in the study design.
Reporting on race, ethnicity, or other socially relevant groupings	There is no population characteristic such as race, ethnicity, or other socially relevant grouping to report in the present manuscript
Population characteristics	There is no population characteristic analysis in the present manuscript
Recruitment	Human skin fibroblasts were collected from neurotypical individuals
Ethics oversight	Skin fibroblasts from all patients were obtained with informed consent approved by the Ethics Committee of the Medical Faculty and the University Hospital Tübingen.

Note that full information on the approval of the study protocol must also be provided in the manuscript.

Field-specific reporting

Please select the one below that is the best fit for your research. If you are not sure, read the appropriate sections before making your selection.

☒ Life sciences ☐ Behavioural & social sciences ☐ Ecological, evolutionary & environmental sciences

For a reference copy of the document with all sections, see nature.com/documents/nr-reporting-summary-flat.pdf

Life sciences study design

All studies must disclose on these points even when the disclosure is negative.

Sample size	Sample size determination is based on the previous experience to obtain significance and reproducibility. No statistical methods were used to predetermine sample sizes. All statistical data resulted from at least 3 independent differentiation onsets with at least 3 technical replicates. The sample sizes were sufficient for statistical analysis and are listed in each figure legend.
Data exclusions	Statistical outlyers were excluded from the analysis using GraphPad Prism (version 10.2.3, RRID: SCR_002798)

Replication	All data resulted from at least 3 independent experiments, the number of independent experiments is indicated in the figure legends.
Randomization	NA. Cells were grouped based in the treatment or the genotype
Blinding	Investigators were blinded to the groups and samples. Investigators involved in data collection of scRNAseq, metabolomics lipidomics and senescent nucleus scores were blinded, investigators performing data analysis were not blinded. The investigators were not blinded because no bias could be made in the analysis, raw data provided for data analysis is publicly available.

Reporting for specific materials, systems and methods

We require information from authors about some types of materials, experimental systems and methods used in many studies. Here, indicate whether each material, system or method listed is relevant to your study. If you are not sure if a list item applies to your research, read the appropriate section before selecting a response.

Materials & experimental systems

n/a	Involved in the study	
☐	☒	Antibodies
☐	☒	Eukaryotic cell lines
☒	☐	Palaeontology and archaeology
☐	☒	Animals and other organisms
☒	☐	Clinical data
☒	☐	Dual use research of concern
☒	☐	Plants

Methods

n/a	Involved in the study	
☒	☐	ChIP-seq
☐	☒	Flow cytometry
☒	☐	MRI-based neuroimaging

Antibodies

Antibodies used

Primary antibodies
Anti-ß-Amyloid 1-16 1/250 Mouse Biolegend 803004 RRID:AB_2715854
ß-Actin 1/1000 Mouse Santa Cruz sc-47778 RRID:AB_626632
ATF5 1/2000 Rabbit Abcam ab184923 RRID: AB 2800462
PECAM-1 (CD31) 1/50-1/500 Mouse Santa Cruz sc-376764 RRID:AB_2801330
CD68 Monoclonal Antibody (KP1) anti-human 1/250 Mouse Thermo Fisher Scientific MA5-13324 RRID:AB_10987212
C1qA 1/1000 Rabbit Proteintech 11602-1-AP RRID: AB_2067153
CTIP2 1/500 Rat Abcam 18465 RRID:AB_2064130
GATA-2 (H-116) 1/100 Rabbit Santa Cruz sc-9008 RRID:AB_2294456
GFAP 1/500 Rabbit Agilent Z0334 RRID:AB_10013382
Histone H2A.X 1/500 Mouse Santa Cruz sc-517348 RRID:AB_2783871
HSP60 (B-9) 1/200 1/2000 Mouse Santa Cruz sc-271215 RRID:AB_10607973
IBA1 (IHC) 1/500 Rabbit Wako 019-19741 RRID:AB_839504
IBA1 1/500-1/1000 Rabbit Wako 016-20001 RRID:AB_839506
LAMP1 1/100 Mouse DSHB H4A3 RRID: AB_2296838
LONP1 1/5000 Rabbit Proteintech 15440-1-AP RRID: AB_2137152
LC3B 1/500 Rabbit Proteintech 18725-1-AP RRID: AB_2137745
MAP2 1/1000-1/5000 Chicken Millipore AB 5622 RRID:AB_91939
OTX2 1/400 Goat Neuromics GT15095 RRID:AB_2157174
p21 WAF1 / CIP1 1/500 Rabbit Cell Signaling Technology 2947S RRID:AB_823586
P62 1/1000 Rabbit Sigma Aldrich P0067 RRID: AB_1841064
PAR 1/500 Mouse AdipoGen Life Sciences AG-20T-0001-C050 RRID:AB_2490281
PAX6 1/500 Rabbit Biolegend 901301 RRID:AB_2565003
PHF Tau Thr181 1/1000 Mouse Thermo Fisher Scientific MN1050 RRID:AB_223651
PITRM1 1/1000 Rabbit Genetex GTX119739 RRID:AB_10617717
pS129 Synuclein 1/1000 Rabbit Genetex GTX50222 RRID:AB_11179523
PSD95-specific, DLG4 1/250 Rabbit Proteintech 20665-1-AP RRID: AB_2687961
Anti-SPI1 Pu.1 (7C6B05) 1/1000 Mouse Biolegend 658002 RRID:AB_2562720
S100ß 1/100 Mouse Sigma Aldrich S2532 RRID:AB_477499
SOX2 1/500 Mouse Abcam ab75485 RRID:AB_1278243
α-synuclein 1/1000 Mouse BD Bioscience 610787 RRID:AB_398108
TBR1 1/500 Rabbit Abcam 31940 RRID:AB_2200219
Human TOMM20 1/1000 Mouse Santa Cruz sc-17764 RRID:AB_628381
Total Tau 1/1000 Mouse Thermo Fisher Scientific MN1000 RRID: AB_2314654
ß-Tubulin 1/500 Mouse Biolegend 802001 RRID:AB_2564645
Vinculin 1/2000 Mouse Santa Cruz sc-73614 RRID:AB_1131294

Secondary antibodies
Anti-Rabbit IgG (H+L) Alexa Fluor™ 488 1/1000 Goat Invitrogen A-11008 RRID:AB_143165
Anti-Rabbit IgG (H+L) Alexa Fluor™ 568 1/1000 Goat Invitrogen A-11011 RRID:AB_143157
Anti-Mouse IgG (H+L) Alexa Fluor™ 488 1/1000 Goat Invitrogen A-11001 RRID:AB_2534069
Anti-Mouse IgG (H+L) Alexa Fluor™ 568 1/1000 Goat Invitrogen A-11004 RRID:AB_2534072

	Anti-Chicken IgG (H+L) Alexa Fluor™ 647 1/1000 Goat Invitrogen A-21449 RRID:AB_2535866<br>Anti -Rat IgG (H+L) Alexa Fluor™ 568 1/1000 Goat Abcam Ab175476 RRID:AB_2813739<br>Anti -mouse IgG, HRP - linked Antibody 1/5000 Horse Cell Signaling Cat# 7076 RRID:AB_330924<br>Anti -rabbit IgG, HRP - linked Antibody 1/5000 Goat Cell Signaling Cat# 7074 RRID:AB_2099233
Validation	All antibodies used in this study have been commercially validated, information is available on the manufacturer's websites

Eukaryotic cell lines

Policy information about cell lines and Sex and Gender in Research

Cell line source(s)	All human iPSC lines used in this study were derived from patients who signed an informed consent form, and the study was approved by the Ethics Committee of the Medical Faculty and University Hospital Tübingen (Ethikkommission der Medizinischen Fakultät am Universitätsklinikum Tübingen). Three healthy control lines were used in the study: two previously generated and characterized healthy donor-derived lines (C1 and C2) following established protocols, and one commercial control line (C3; Kolf2.1J, RRID:CVCL_B5P3; Jackson Laboratory, Bar Harbor, USA). The PITRM1-knockout lines were previously generated from C2.
Authentication	iPSC cell lines were routinely assesed by Sanger sequencing for cell identity.
Mycoplasma contamination	All cell lines were routinely assessed for mycoplasma using VenorGeM Classic kit (Minerva Biolabs). All cell lines tested negative for mycoplasma contamination
Commonly misidentified lines<br>(See ICLAC register)	There are no commonly misidentified cell lines

Animals and other research organisms

Policy information about studies involving animals; ARRIVE guidelines recommended for reporting animal research, and Sex and Gender in Research

Laboratory animals	The C57BL/6n-Atm1Brd Pitrm1+/- mice used in this study were kindly provided by the Sanger Institute (http://www.informatics.jax.org/allele/MGI:5085349).
Wild animals	No wild animals were used in this study
Reporting on sex	The experimental design included two groups of female mice of 6 and 15 months of age (6 Pitrm1+/+ vs. 6 Pitrm1+/-).
Field-collected samples	Animals were housed three per cage with room temperature set at 22 ± 2°C and a 12-hour light-dark cycle and 60% relative humidity, with ad libitum access to food (Rodents standard diet catalog number 4RF25, Mucedola, Settimo Milanese, Milan, Italy) and water.
Ethics oversight	All experiments were performed in accordance with the European Community Council Directive 2010/63/EU. According to current Italian law (D. Lgs. n. 26/2014), the protocol was approved by the General Direction of Animal Health and Veterinary Drugs of the Italian Ministry of Health with the authorization number 483/2023-PR

Note that full information on the approval of the study protocol must also be provided in the manuscript.

Plants

Seed stocks	*Report on the source of all seed stocks or other plant material used. If applicable, state the seed stock centre and catalogue number. If plant specimens were collected from the field, describe the collection location, date and sampling procedures.*
Novel plant genotypes	*Describe the methods by which all novel plant genotypes were produced. This includes those generated by transgenic approaches, gene editing, chemical/radiation-based mutagenesis and hybridization. For transgenic lines, describe the transformation method, the number of independent lines analyzed and the generation upon which experiments were performed. For gene-edited lines, describe the editor used, the endogenous sequence targeted for editing, the targeting guide RNA sequence (if applicable) and how the editor was applied.*
Authentication	*Describe any authentication procedures for each seed stock used or novel genotype generated. Describe any experiments used to assess the effect of a mutation and, where applicable, how potential secondary effects (e.g. second site T-DNA insertions, mosiacism, off-target gene editing) were examined.*

Flow Cytometry

Plots

Confirm that:

☒ The axis labels state the marker and fluorochrome used (e.g. CD4-FITC).

☒ The axis scales are clearly visible. Include numbers along axes only for bottom left plot of group (a 'group' is an analysis of identical markers).

☒ All plots are contour plots with outliers or pseudocolor plots.

☒ A numerical value for number of cells or percentage (with statistics) is provided.

Methodology

Sample preparation	For cell cycle analysis, iPSC-derived neurons, astrocytes and microglia were detached with Accutase and stained in suspension with Hoechst 33342 (2.5 µg/ml) for 30 mins at 37°C. Cells were then washed and stained with Zombie NIR viability dye (1:1000) for 30 minutes at room temperature. Cells were washed and kept in 2% FBS in PBS until reading with flow cytometer. For phagocytosis measurements, microglia were incubated for 3 hours at 37°C with pre-opsonized Alexa 488 latex beads. Cells were washed with PBS, detached with Accutase and stained with Zombie NIR as described above. For cytokine measurements, supernatants were incubated with LEGENDplex™ bead panels according to the manufacturer's protocol.
Instrument	FACS experiments were either performed with SonyID7000 Spectral Cell Analyzer (Sony Biotechnology, San Jose, USA) or Acea NovoCyte® Flow Cytometer (Agilent Technologies, Santa Clara, USA). The SonyID7000 Spectral Cell Analyzers is equipped with 5 lasers. The Acea NovoCyte® Flow Cytometer is equipped with 3 lasers.
Software	Data was collected with respective instrument software (Sony ID 7000 system software, Agilent NovoExpress Software). Collected data was exported as fcs. files followed by analysis with FlowJoTM software (BD Biosciences, Version 10.10) or LEGENDplexTM software (BioLegend).
Cell population abundance	For cell death measurements, more than 100 000 cells were analysed (Supplementary Figure 3). For cell cycle analysis, more than 5 000 Hoechst 33342-positive cells were used as input for cell cycle module in FlowJoTM software (Supplementary Figure 5). For cytokine measurements, more than 300 beads were recorded and analysed for each target cytokine using LEGENDplexTM software.
Gating strategy	Gating strategy for analysis of LegendPlex assays followed automated gating strategy from LEGENDplexTM software. For cell death, cell cycle or phagocytosis measurements, cell debris was removed based on FSC-A versus SSC-A. Single cells were gated using FSC-A versus FSC-H. Live cells were selected based on FSC-A versus Zombie NIR-A. For cell cycle analysis, Hoechst 33342-positive cells were selected and used as input for cell cycle analysis tool. G1, S and G2/M peaks were assigned using the cell cycle module and Watson pragmatic model. Analysis with same settings and boundary positions were applied to all samples within same experiment and cell type. For phagocytosis analysis, cells were separated into phagocytosing and non-phagocytosing cells by gating on AF488-A versus FSC-A. Positive and negative boundaries for each staining were set using unstained and single-stained controls.

☒ Tick this box to confirm that a figure exemplifying the gating strategy is provided in the Supplementary Information.
